# Gut dysbiosis mediates the association between antibiotic exposure and chronic disease

**DOI:** 10.3389/fmed.2024.1477882

**Published:** 2024-11-06

**Authors:** Francisco Guarner, Luis Bustos Fernandez, Sylvia Cruchet, Adérson Damião, Aldo Maruy Saito, Juan Pablo Riveros Lopez, Luciana Rodrigues Silva, Miguel Angel Valdovinos Diaz

**Affiliations:** ^1^Centro Medico Teknon, Barcelona, Spain; ^2^Centro Medico Bustos Fernandez, Instituto de Gastroenterologia, Buenos Aires, Argentina; ^3^Institute of Nutrition and Food Technology, Universidad de Chile, Santiago, Chile; ^4^Department of Gastroenterology, University of São Paulo School of Medicine, São Paulo, Brazil; ^5^Catedra de Pediatria, Hospital Cayetano Heredia, Universidad Peruana Cayetano Heredia, Lima, Peru; ^6^Department of Pediatric Gastroenterology, El Bosque University, Bogotá, Colombia; ^7^Pediatric Gastroenterology Service, Federal University of Bahia, Salvador, Brazil; ^8^Servicio de Gastroenterología, Hospital Médica Sur, Ciudad de México, Mexico

**Keywords:** antibiotics, probiotics, DOHaD, long-term, dysbiosis, chronic disease, neuroinflammation, SCFA

## Abstract

Antibiotics are safe, effective drugs and continue to save millions of lives and prevent long-term illness worldwide. A large body of epidemiological, interventional and experimental evidence shows that exposure to antibiotics has long-term negative effects on human health. We reviewed the literature data on the links between antibiotic exposure, gut dysbiosis, and chronic disease (notably with regard to the “developmental origins of health and disease” (“DOHaD”) approach). Molecular biology studies show that the systemic administration of antibiotic to infants has a rapid onset but also often a long-lasting impact on the microbial composition of the gut. Along with other environmental factors (e.g., an unhealthy “Western” diet and sedentary behavior), antibiotics induce gut dysbiosis, which can be defined as the disruption of a previously stable, functionally complete microbiota. Gut dysbiosis many harmful long-term effects on health. Associations between early-life exposure to antibiotics have been reported for chronic diseases, including inflammatory bowel disease, celiac disease, some cancers, metabolic diseases (obesity and type 2 diabetes), allergic diseases, autoimmune disorders, atherosclerosis, arthritis, and neurodevelopmental, neurodegenerative and other neurological diseases. In mechanistic terms, gut dysbiosis influences chronic disease through direct effects on mucosal immune and inflammatory pathways, plus a wide array of direct or indirect effects of short-chain fatty acids, the enteric nervous system, peristaltic motility, the production of hormones and neurotransmitters, and the loss of intestinal barrier integrity (notably with leakage of the pro-inflammatory endotoxin lipopolysaccharide into the circulation). To mitigate dysbiosis, the administration of probiotics in patients with chronic disease is often (but not always) associated with positive effects on clinical markers (e.g., disease scores) and biomarkers of inflammation and immune activation. Meta-analyses are complicated by differences in probiotic composition, dose level, and treatment duration, and large, randomized, controlled clinical trials are lacking in many disease areas. In view of the critical importance of deciding whether or not to prescribe antibiotics (especially to children), we suggest that the DOHaD concept can be logically extended to “gastrointestinal origins of health and disease” (“GOHaD”) or even “microbiotic origins of health and disease” (“MOHaD”).

## 1 Introduction

**Figure 1:**
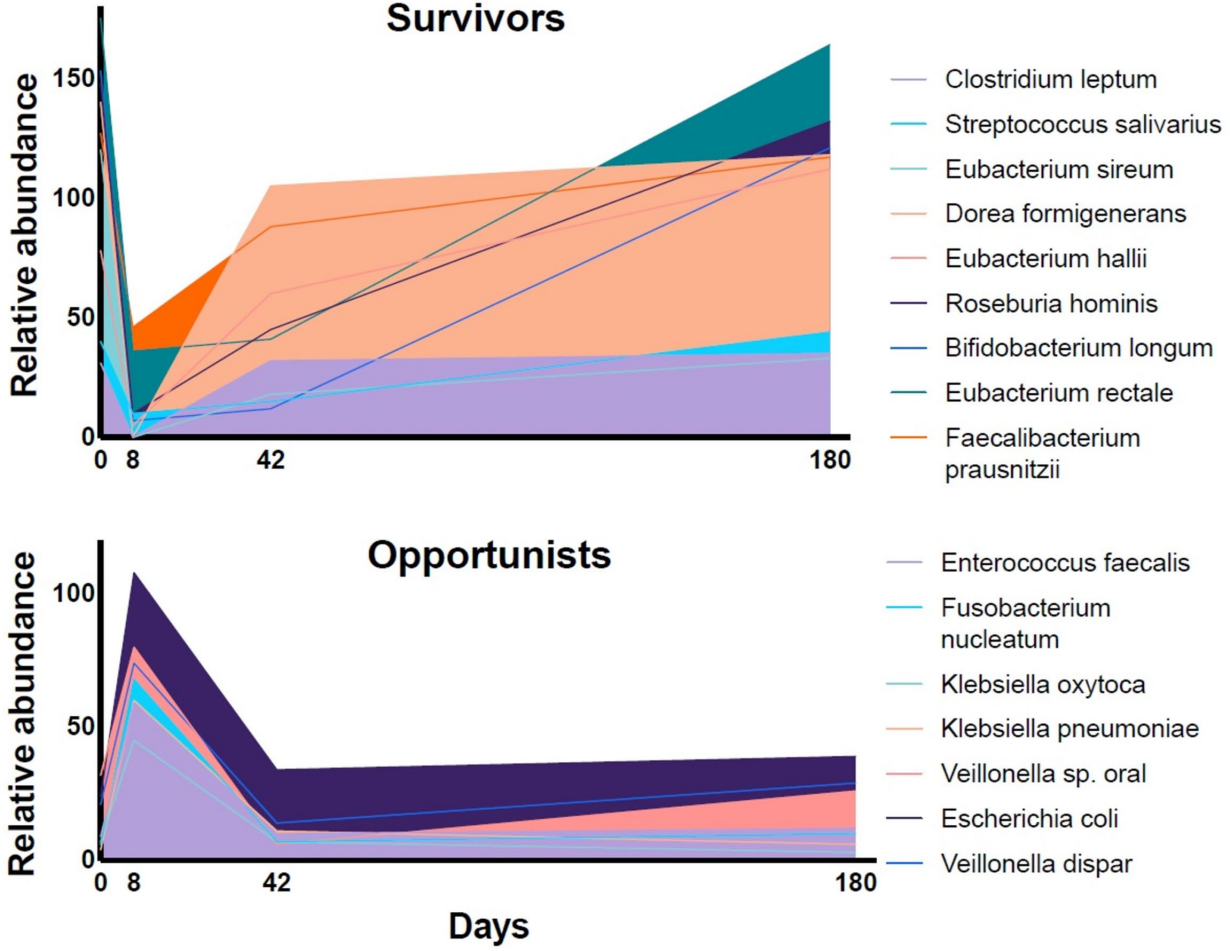
The relative abundance of representative species in fecal samples of volunteers during a 6-month follow-up period after a four-day course of a combination of meropenem, gentamicin, and vancomycin [data extracted from Palleja et al. (11)]. The relative abundance of the dominant fermentative species (“Survivors”) dropped during the antibiotic treatment and recovered slowly over the follow-up period. The relative abundance of subdominant, resistant bacteria (“Opportunists”) increased rapidly.

Worldwide, antibiotics continue to save millions of lives, relieve suffering, and prevent long-term illness (1). Large quantities of these drugs are involved: for example, over 250 million prescriptions of antibiotics were issued in the USA alone in 2016 (2). One can reasonably hypothesize that worldwide, most people (especially children) are treated with an antibiotic at least once a year (3–6). Antibiotics are extremely effective and generally lead to the eradication of the targeted pathogenic bacteria (7). By definition, antibiotics create dysbiosis [defined in several ways (8)] by killing significant components of the gut community (9–11) (Figure 1). Broadly, levels of *Enterobacteriaceae*, *Bacteroidaceae*, enterococci, and drug-resistant *Escherichia coli* rise following antibiotic treatment in adults, whereas levels of bifidobacteria, lactobacteria, actinobacteria and *Lachnospiraceae* decrease (11–13). Despite the emergence of microbial resistance as a long-term public health risk, antibiotics are still irreplaceable in the treatment of bacterial infections (14).

However, antibiotics also have negative effects on health. The acute (short-term) effects have been extensively studied; for example, between 5 and 20% of antibiotic users (depending on the population studied) will develop antibiotic-associated diarrhea with days or weeks of treatment initiation, and the incidence is greatest in frail, hospitalized patients and young children (11, 15–19). There is now a large body of epidemiological evidence to show that exposure to antibiotics has chronic (long-term) negative effects on human health—effects likely to be accentuated by poor antibiotic stewardship, inappropriate prescribing, excessive or chronic administration, and off-label use.

The present review covers the literature data on the long-term negative health effects of antibiotic exposure, the link to dysbiosis, and how the associated risks might be managed through the evidence-based administration of specific probiotics. Although this review was not systematic, we searched the PubMed database for recent publications (from January 1st, 2023, to June 15th, 2024) using logical combinations of the following keywords: antibio*, exposure, microbiot*, microb*, gut, intestine*, dysbiosis, DOHaD, thousand days, health, disease, and probiotic.

## 2 The gut microbiota and its maturation

From the mouth to the anus, the adult human gastrointestinal tract has a surface area of around 30 m^2^ and a luminal volume of around 3 L (20). After the skin, the gastrointestinal tract constitutes the body’s largest interface with the environment. The gut carries 10^13^–10^14^ microbes from thousands of species, which contain several million genes in total—far more than in the human genome. Here, we shall use the term “gut microbiota” to refer to the set of microbial (mainly bacterial) species contained in the environment of the human gastrointestinal tract. We prefer “microbiota” to the term “microbiome,” which we take to encompass not only the microorganisms (bacteria, archaea, and lower and higher eukaryotes) and their genomes but also the human cells and substances in the surrounding gut (21). Although there are huge inter- and intra-individual variations in the composition of the gut microbiota, over 99% of the gut microbiota is composed of species from the Firmicutes, Bacteroidetes, Proteobacteria, and Actinobacteria (22).

It is generally accepted that a human’s gut microbiota starts to form after birth (i.e., *ex utero*) and reaches maturity at around the age of 3 years (23, 24). Interesting, this period coincides largely with the “first 1,000 days” (from conception to the age of 2 years)? The “first 1,000 days” concept grew out of a body of research on the importance of the early life environment for the child’s current and future mental, physical and emotional health states (25–30). Awareness of this concept grew markedly after a keynote speech by the then US Vice President Hilary Clinton at the “1,000 Days: Change a Life, Change the Future” international conference on global child undernutrition in 2010. Furthermore, the “first 1,000 days” concept fits well with the “thrifty phenotype” hypothesis, the Barker hypothesis (linking adverse nutrition childhood to metabolic syndromes in adulthood) and the subsequent “developmental origins of health and disease” (DOHaD) concept; all hold that a poor fetal environment and perturbed early neonatal life prompt the development of disease (31–33). Of course, a focus on the first 1,000 days does not mean that day 1,001 is medically and scientifically unimportant. Nevertheless, the “first 1,000 days” concept has been useful for (i) focusing research efforts and public opinion on this critical period in the development of the child and, indeed, of the microbiota, and (ii) emphasizing the importance of the microbiota’s origin and development in the first hours, days, weeks, months and then years of the human host’s life.

As mentioned above, most experts consider that the human gut microbiota starts to form after birth. However, there is some debate as to the presence of a fetal microbiota, and it is not inconceivable that bacteria from the mother can cross the maternal-fetal barrier (34). However, the ethical and practical difficulties of collecting contamination-free samples of fetal tissue have complicated research efforts to settle this debate (35–39). Delivery by cesarean section (i.e., the avoidance of contact with the vaginal microbiota) is a major dysbiosis-promoting factor and is associated with a low-*Bacteroides* profile in the first 6 months postpartum (40). In children delivered by cesarean section, relatively high abundances of *Burkholderiaceae*, *Bacteroidales*, and *Ruminococcaceae* persist for at least 7 years (41, 42). It is also noteworthy that the gut microbiota is much less diverse in preterm infants (born after less than 34 weeks of gestation) than in term infants—even in the absence of antibiotic treatment, which most preterm infants nevertheless receive. Cetinbas et al. used 16S rDNA sequencing to evaluate long-term, antibiotic-induced dysbiosis on the basis of 363 stool samples collected between 26 and 48 weeks of adjusted age from 65 preterm infants treated with antibiotics in the NICU and 52 samples from 14 preterm counterparts not treated with antibiotics (43). Antibiotic-treated preterm infants were slower to return to a “normal” gut microbiota, in terms of Shannon diversity and species richness. The difference in the abundance of *Paenibacillus amylolyticus* was quickest to disappear, whereas changes in *Veillonella*, *Shuttleworthia* and *Clostridioides* persisted up to 40 weeks of age (43).

During the potentially unstable first years of life, the gut microbiota can be perturbed by pathogenic microbes and external (environmental) factors, such as diet, xenobiotics, and other exogenous compounds—notably antibiotics (44). In-depth molecular biology studies have shown very clearly that the systemic administration of an antibiotic to infants has a rapid but often long-lasting impact. For example, Yassour et al.’s study of 1,069 samples of feces from 39 children (around half of whom had been treated with antibiotics in the first year of life) collected over a period of 36 months showed that antibiotic-treated individuals had less stable gut microbiotas, with low diversity still visible at the age of 3 years (40). Kwon et al. used 16S rRNA sequencing and linear discriminant effect size analysis to evaluate alpha and beta diversities in fecal samples from (i) 20 infants under 3 months of age who had received antibiotics for at least 3 days and (ii) 34 age-matched, healthy controls not exposed to antibiotics (45). Relative to controls, the relative abundances of *Escherichia*, *Shigella* and *Bifidobacterium* were significantly greater in the antibiotic-treated group, whereas that of *Bacteroides* was significantly lower. The abundances of *Firmicutes* genera (*Allobaculum*, *Enterococcus*, and *Candidatus arthromitus*), *Proteobacteria* genera (*Klebsiella*), and *Actinobacteria* genera (*Bifidobacterium*) were three or four times higher in the treated group than in the control group. A phylogenetic investigation of communities by reconstruction of unobserved states revealed a significant difference in gut microbiome metabolic activity between the two groups; the antibiotic-treated group showed the expression of significantly more genes involved in naphthalene degradation, glycolysis gluconeogenesis, and lipoic acid metabolism and less expression of genes involved in porphyrin metabolism and fatty acid biosynthesis (45).

The effects of antibiotics on the gut microbiota of newborns and infants are accentuated by a lack of standardization in antibiotic treatment plans. For example, Schulman et al.’s study of 127 neonatal intensive care units (NICUs) in the USA evidenced up to 40-fold variations in dosing regimens (46). In France, Leroux et al. study of 44 NICUs found an average of nine different dosing regimens for each of the 41 antibiotics documented (47). Similarly, in 43 NICUs in the UK, Kadambari et al. identified 10 different dosing regimens for prescriptions of gentamicin (48).

## 3 “Normal,” “healthy” microbiotas: how can dysbiosis be defined?

Although many experts have sought to define dysbiosis, the topic is subject to debate and constitutes a field of research in its own right (49). As emphasized by Brüssow and by Drago et al., no consensus conference has yet worked out a definition of “dysbiosis” (50, 51). To the best of our knowledge, none of the learned societies or medical associations in this field have issued a single, unambiguous definition of “dysbiosis.” Given this lack, there is no “gold standard” approach to identifying a dysbiotic state (52). A conventional, broad definition published by Petersen and Round in 2014 is related primarily to the composition of the microbiota: “any change to the composition of resident commensal communities relative to the community found in healthy individuals” (13). These compositional changes usually encompass a decrease in bacterial diversity, the loss of beneficial microbes, and overgrowth by potential pathogens. More recent definitions have emphasized function as well as composition: for example, Levy et al. define dysbiosis as “a compositional and functional alteration in the microbiota that is driven by a set of environmental and host-related factors that perturb the microbial ecosystem to an extent that exceeds its resistance and resilience capabilities” (53). In this definition, gut microbiota can be viewed as a conceptual energy landscape in which both healthy and dysbiotic states can exist in energy minima. External factors (such as antibiotics, various xenobiotics, dietary components, and pathogens) that exceed a certain threshold cause transitions from a stable, healthy state to a metastable, dysbiotic state (54). Levy et al. acknowledged that given the high degree of interindividual variability, it is impossible to define a single “healthy” microbiota; however, they suggest that the main characteristic of a “healthy” microbiota in a given individual is richness and stability over time (53). Wilkins et al. have suggested that dysbiosis should be defined by reference to a particular disease state, i.e., a single, all-encompassing definition may not be feasible (49). Lastly, Malard et al. have defined dysbiosis as the disruption of host-microbe symbiosis and crosstalk, with auto-aggravating signals from both the host and microbes that maintain the metastable dysbiotic state (54). Hence, as detailed in the following section, dysbiosis of the gut microbiota can be viewed as the disruption of the positive actions of (i) short-chain fatty acids (SCFAs, produced by the fermentation of host-enzyme-resistant carbohydrates by obligate anaerobes) (55, 56), (ii) the microbial production of serotonin, catecholamines and other neurotransmitters (affecting gut peristaltic motility and the genesis of the enteric nervous system) (57–61), (iii) minimization of the “leaky gut” in which potentially pathogenic whole bacteria and bacterial wall components can enter the circulation (62–64), and (iv) the bacterial expression of enzymes that influence the levels of host metabolites (65, 66). In summary, dysbiosis does not have a consensus definition but can usefully be viewed as the disruption of a previously stable, symbiotic, functionally complete microbiota by dietary, xenobiotic or other factors.

## 4 Mechanistic links between the microbiota, dysbiosis, health, and chronic disease

It is now clear that a stable, functionally rich gut microbiota exerts complex direct and indirect effects on the human host’s health through multiple genetic, immune-mediated and metabolic factors. Conversely, dysbiosis of the gut microbiota (i.e., the disruption of a previously stable, functionally complete microbiota) has many harmful short- and long-term effects on health. Common signs of gut dysbiosis include diarrhea, gas, constipation, nausea, and even chest pain (67). Admittedly, in most settings, it is difficult to determine whether dysbiosis is the cause or result of disease. Determining the causal nature and direction of a relationship notably requires prospective, longitudinal studies to see whether the dysbiosis or the disease occurs first. However, this approach can be confounded if a patient remains free of symptoms for months or years. A causal role of antibiotic-associated gut dysbiosis can nevertheless be suspected when the disease is more intense after exposure to broad-spectrum antibiotics (compared with narrow-spectrum antibiotics) and shows an antibiotic dose dependence (68–70). Lastly, one cannot rule out direct, negative effects (i.e., not mediated by the gut microbiota) of antibiotics. Some classes of antibiotic affect myocytes and neurons directly; for example, aminoglycosides, capreomycin, and macrolides are ototoxic (71, 72). Below, we describe the main pathways through which the gut microbiota are known to influence human health.

### 4.1 Short-chain fatty acids

The SCFAs [acetic (C2), propionic (C3), butyric (C4), and valeric (C5) acids] are produced via the fermentation of host-enzyme-resistant carbohydrates by obligate anaerobes (primarily members of the Bacteroidetes and Firmicutes, including *Roseburia* spp., *Prevotella* spp., *Ruminococus* spp., *Coprococcus* sp., *Akkermansia muciniphila*, *Faecalibacterium prausnitzii*, and *Eubacterium rectale*), in collaboration with bifidobacteria (73). SCFAs are produced at up to millimolar concentrations in the gut and have several distinct metabolic and regulatory effects on the host.

Firstly, some of the SCFAs’ extracellular actions are exerted through via the G-protein-coupled free fatty acid receptors 2 and 3 (FFA2 and FFA3), which are involved in responses to immune challenges (74, 75). Secondly, acetate and propionate bind to the G-protein-coupled olfactory receptor 51E2 (found notably in intestinal and enteroendocrine tissues) and the aryl hydrocarbon receptor (76–79). Thirdly, butyrate is a competitive inhibitor of histone deacetylase, which leads to the hyperacetylation of histones, increases chromatin accessibility and has major epigenetic effects on gene transcription (55, 56). Importantly, butyrate’s inhibition of histone deacetylase promotes histone acetylation in the Foxp3 gene’s promoter and enhancer regions in naïve T-cells in the colon and promotes their differentiation into peripheral regulatory T cells (key players in the immune tolerance of antigens during development) (80, 81). Hence, a low level of butyrate in the intestinal tract (due to antibiotic-induced dysbiosis) impairs Treg differentiation and the immune system’s ability to suppress excessive immune responses, which in turn may lead to mucosal and systemic inflammatory states. Lastly, the low oxygen levels required by the colon-dwelling strict anaerobes associated with a health microbiota are maintained by the *β*-oxidation of bacterially produced butyrate. Indeed, butyrate is the healthy colonocyte’s main energy substrate, accounting for 70–80% of its energy needs (82). A lack of SCFA-producing bacteria will tend to increase oxygen levels in the gut lumen and thus favor the expansion of aerobes and facultative anaerobes with pro-inflammatory potential.

### 4.2 The enteric nervous system and gastrointestinal dysmotility

The enteric nervous system (ENS) is now acknowledged to be a complex network of neurons and enteric glial cells that controls gastrointestinal motility, blood flow, and immune responses. The gut microbiota’s direct or indirect influences on the ENS are signaled to the brain through vagal afferents (83). Conversely, the brain’s responses to stress may be passed to the ENS by vagal efferents (83). The gut microbiota can affect the genesis of the ENS and gut peristaltic motility by producing serotonin (57, 58). Studies in mice have shown that the administration of antibiotics modified the gut microbiome (with marked decreases in the abundance of *Clostridioides*, *Lachnoclostridium*, and *Akkermansia*), induced intestinal dysmotility, and interfered with the differentiation of myenteric neurons and the expression of the neuronal marker beta III-tubulin in the myenteric plexus (84, 85). These and other observations indicate that antibiotic-induced gut dysbiosis is associated with structural and functional damage to the ENS, including gastrointestinal dysmotility.

### 4.3 Catecholamines and neurotransmitters

Some gut microbial strains produce and/or respond to compounds that serve as hormones, neurotransmitters or their precursors in the human host, including catecholamines, gamma aminobutyric acid (produced by lactobacilli and bifidobacteria), acetylcholine (lactobacilli), dopamine (*Bacillus* sp.), neuropeptides, noradrenaline (*Bacillus* sp.), serotonin (*Escherichia*, *Enterococcus*, and *Streptococcus*), endocannabinoids, histamine, and tryptophan (*bifidobacteria*) (57, 59–61). The gut microbiota can thus be said to have a direct action on the nervous system.

### 4.4 Intestinal barrier integrity, immune signaling, and inflammation

The gut microbiota helps to protect the intestinal mucosa—notably through the presence of mucus-promoting commensals and (in the colon) the supply of butyrate as an energy substrate to colonocytes (86). Dysbiosis can trigger the loss of intestinal barrier integrity. The resulting passage of pathogenic and non-pathogenic microbes leads to antigen presentation and the activation of innate and adaptive immune cells. One of the key pro-inflammatory signals in the loss of intestinal barrier integrity is the endotoxin lipopolysaccharide from the cell wall of Gram-negative bacteria (87). Lipopolysaccharide induces inflammation by activating Toll-like receptor 4 and upregulating nuclear factor-kappa B (88).

### 4.5 Dietary induction of enzymes

The composition of the diet influences the expression of a wide range of bacterial enzymes (cholesterol dehydrogenase, beta glucosidase and glucuronidase, 7-alpha-hydroxylase, nitroreductase, and azoreductase), notably by bacteria in the colon (65, 66). In turn, the activity of these enzymes influences levels of key host metabolites.

## 5 Chronic disease, environmental factors, antibiotics, and dysbiosis

Many chronic diseases are known to have both genetic factors and environmental susceptibility/triggering factors, of which antibiotic exposure is only one. Many of these environmental factors are linked to a “modern,” “post-industrial” or “Western” lifestyle, including the “Western diet” (high in sugar, fat, and processed food components, and low in fiber) and sedentary behavior (typically defined as spending time in a sitting, reclining or lying posture with an energy expenditure of 1.5 metabolic equivalents of task or less) (89–91). Sedentary behavior (including total sitting time and TV viewing time) is associated with an elevated risk of chronic diseases, including diabetes, cardiovascular disease, and (to a lesser extent) cancer—even after adjustment for the level of physical activity (92). Interestingly, lower sedentary behavior through increased physical activity is known to influence gut microbiota diversity in adults and children, with higher alpha-diversity (notably including SCFA-producing *Lachnospiraceae* and *Erysipelotrichaceae* families and *Akkermansia*, *Roseburia*, and *Veillonella*, genera) in physically active individuals (93–96). Indeed, SCFAs appear to be the main molecular link between physical activity and the gut microbiome, although few published studies of physically active vs. sedentary populations controlled for dietary confounders (i.e., fiber intake) (97). The high-sugar, high-fat “Western diet” is clearly associated with dysbiosis, as characterized by a decrease in the abundance of Bacteroidetes and bifidobacteria and an increase in organisms that can utilize excess monosaccharides, such as Enterobacteriaceae and Proteobacteria (98–103). Again, the main molecular link between diet, a normal microbiota and the associated health effects on the host is likely to be the SCFAs (104).

**Figure 2:**
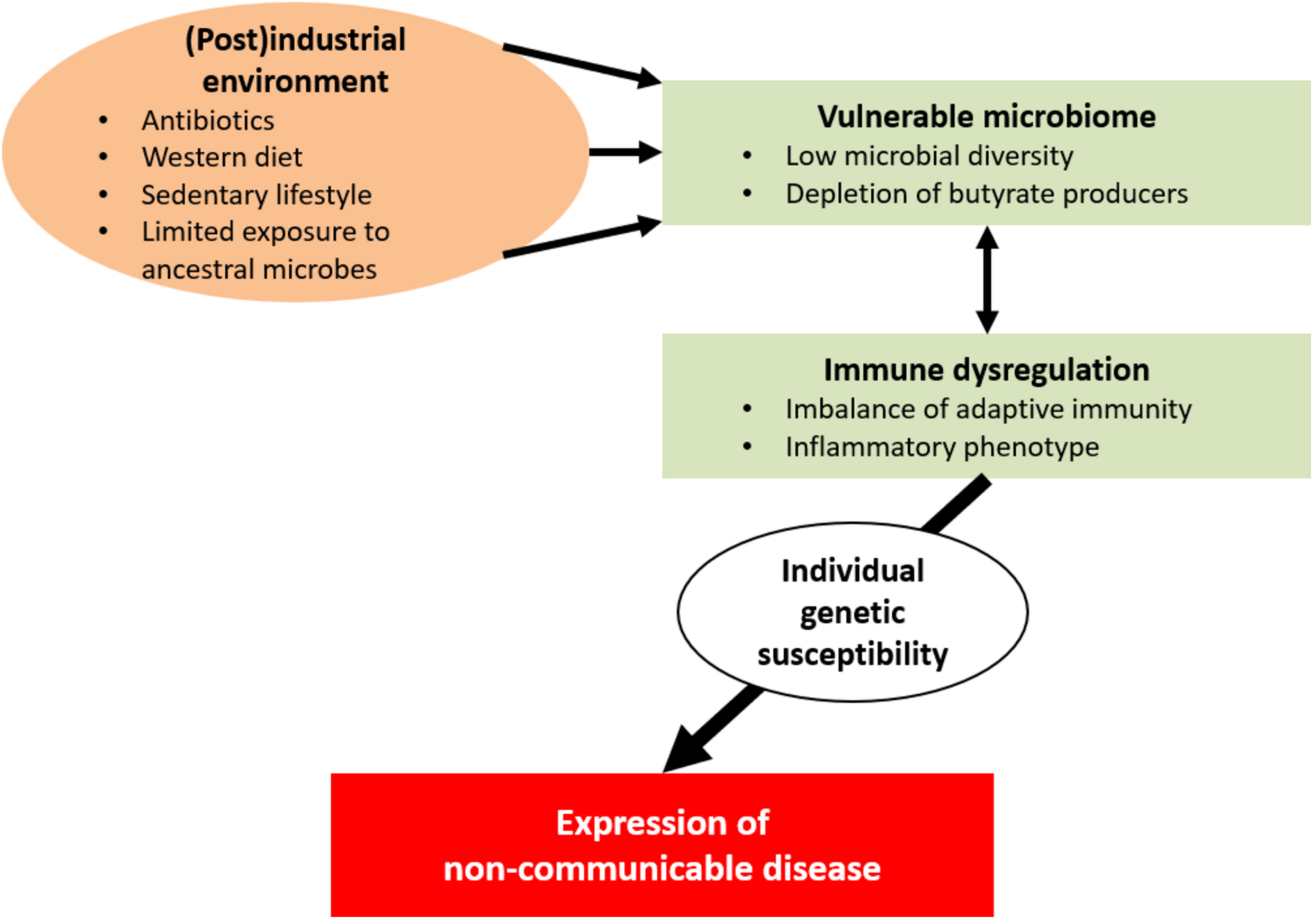
In industrial and postindustrial societies, a number of environmental factors have detrimental consequences for a vulnerable human microbial ecosystem; these factors include exposure to antibiotics and xenobiotics, sanitation of the living space, Western-type diets, sedentariness, and pollution. This unwanted alterations to the gut microbiome appear to be suboptimal for human health. Interactions between the altered microbiome and the host’s immune system contribute to the development of immune dysregulation and inflammatory phenotypes. In turn, these disturbances may lead to chronic inflammation and tissue injury. Individual genetic susceptibility might eventually determine the clinical expression of non-communicable diseases (e.g., inflammatory, autoimmune, metabolic and neoplastic conditions and cognitive disorders).

Antibiotic-induced perturbation of the gut microbiome nevertheless constitutes a key “environmental” factor in the development of chronic disease (Figure 2). The prevalent use of antibiotics in infants reveals concerning associations between antibiotic exposure and the onset of a number of distinct immunological, metabolic and neurobehavioral health conditions (whether isolated or combined) during childhood. For example, in a time-to-event analysis of medical records for 14,572 children born in Olmsted County (MI, United States) between January 1, 2003, and December 31, 2011, Aversa et al. found that antibiotic exposure in the first 2 years of life (i.e., during the first 1,000 days) was associated with asthma, allergic, rhinitis, atopic dermatitis, celiac disease, overweight, obesity, attention deficit hyperactivity disorder, and learning disability (105). These health risks were influenced by the number, type, and timing of the antibiotic prescriptions. Relationships between antibiotics, dysbiosis, and a number of chronic diseases are described below.

### 5.1 Inflammatory bowel disease

Inflammatory bowel disease (IBD) primarily encompasses Crohn’s disease (CD) and ulcerative colitis (UC). Fecal samples from people with IBD (especially CD) are characterized by a low abundance of Firmicutes and a high abundance of Proteobacteria (106). Interestingly, people with IBD also have an abnormally high abundance of the mucin-degrading bacteria *Ruminococcus gnavus* and *Cenarchaeum symbiosum*, even though these species are present in the healthy gut (106).

A large body of evidence suggests that gut dysbiosis [alone or in combination with other factors, such as diet, smoking, pollution, xenobiotics, and genetic factors causing a leaky gut (i.e., increased permeability)] may cause gut inflammation and thus IBD (107). The gut mycobiome and gut virome have been implicated in this dysbiosis, along with the bacterial communities of the gut microbiota (107–109). It has been suggested that dysbiosis early in life leads to CD, whereas dysbiosis at any time in life contributes to the onset of UC (110).

Ungaro et al. meta-analysis of 11 observational studies (including 7,208 children and adults diagnosed with IBD) found that exposure to antibiotics increased the risk of being newly diagnosed with CD (odds ratio (OR) [95% confidence interval (CI)]: 1.74 [1.35–2.23]) but not ulcerative colitis (1.08 [0.91–1.27]) (111). However, the data are contradictory: Troelson and Jick’s case–control study of 461 patients with UC and 683 patients with CD did not find any association with prior antibiotic use (112). A nation-wide observational study in Denmark confirmed the association between antibiotic exposure and elevated, independent risks of CD and UC. A positive dose–response relationship was also observed: the higher the level of exposure, the greater the risk (68).

It has been hypothesized that the effects of dysbiosis in IBD are mediated through SCFAs, the mechanistic roles of which have been outlined above. Holota et al. found that a 14-day course of ceftriaxone treatment in male Wistar rats led to a greater caecum weight, a fall in SCFA levels, the sustained elevation of conditionally pathogenic enterobacteria such as *E. coli*, *Clostridioides*, *Staphylococcus* spp. and hemolytic bacteria, increased colonic epithelial permeability, greater bacterial translocation, and lower levels of FFA2 and FFA3 receptors and SMCT1 and higher levels of MCT1 and MCT4 SCFA transporters in the colonic mucosa. Importantly, the ceftriaxone-treated animals were more susceptible to experimental colitis (113).

### 5.2 Irritable bowel syndrome

Although irritable bowel syndrome (IBS) does not feature gut inflammation and tissue damage, there is evidence of (i) dysbiosis in people with this functional gastrointestinal disorder (FGID) and (ii) an association between antibiotic use and the development of FGIDs. Saffouri et al. reported that the microbial composition of the small intestinal was significantly altered in symptomatic patients with IBS, with lower phylogenetic alpha diversity, richness, and evenness, and significant decreases in the abundances of *Porphyromonas*, *Prevotella*, and *Fusobacterium* (114). Jones et al. retrospectively studied electronic medical records from over 15,000 patients seen in general practice in the UK. Antibiotics were prescribed more frequently to patients with one or more FGIDs than to healthy individuals. A significant minority (7–14%) of individuals with an FGID received their first recorded antibiotic in the 12 months prior to the FGID diagnosis (115).

### 5.3 Celiac disease

People with active celiac disease have dysbiosis, with greater abundances of *Enterobacteriaceae*, *Proteobacteria*, *Staphylococcaceae*, and *Proteobacteria* (70). Furthermore, a reduced abundance of *Streptococcus mutans* and *Streptococcus anginosus* was observed in patients with active celiac disease patients and also in those with nonactive disease. Sander et al.’s analysis of data from a register-based cohort study conducted in Denmark from 1995 to 2012 and in Norway from 2004 to 2012 showed that exposure to systemic antibiotics in the first year of life was associated in a dose-dependent manner with the diagnosis of celiac disease (OR [95%CI]: 1.26 [1.16–1.36]) (116), although further research on this topic is required. People with celiac disease should consume a gluten-free diet throughout their life: however, the latter is associated with a microbiota characterized by low abundances of *Bifidobacterium* sp. and *Lactobacillus* sp., and higher abundances of pathobionts like *E. coli* and the Enterobacteriaceae (117).

### 5.4 Neurological, neurodevelopmental, and neurodegenerative diseases

The increase in the prevalence of multifactorial neurodevelopmental diseases (NDDs, including autism spectrum disorder and attention deficit-hyperactivity disorder) over the last few decades suggest that the prevalence of environmental triggering and/or susceptibility factors (such as antibiotic exposure) during prenatal, perinatal, and postnatal time windows has also increased. Dysbiosis is a known feature of NDDs; individuals with these conditions, notably have a higher fecal abundance of *Bacterioidetes* and *Megamonas,* and a lower abundance of bifidobacteria, *Veillonella*, *Escherichia*, *Ruminococcaceae*, *Streptococcaeceae*, *Peptostreptococcaceae*, and *Erysipelotrichaceae* (118–120).

Population-based studies have highlighted a clear association between antibiotic exposure and the risk of neurodegenerative diseases. For example, Kim et al. conducted a retrospective study of claims data in a Korean nationally representative cohort (*n* = 313,161 participants) (69). After adjustments for covariates, used of antibiotics for 91 or more days over the period from 2002 to 2005 had an elevated risk of dementia in general (adjusted hazard ratio [95%CI] = 1.44 [1.19–1.74]), Alzheimer’s disease (AD: 1.46 [1.17–1.81]) and vascular dementia (1.38 [0.83–2.30]) during the follow-up period from 2006 to 2013. The researchers noted a dose dependency; people having received five or more classes of antibiotic during the study period had higher risks of dementia and AD (but not vascular dementia) (69). A large body of epidemiological research has linked the use of antibiotics during pregnancy (for the treatment of maternal infections) in particular with an elevated risk of NDDs and of cognitive disorders in adulthood (121, 122). The risk appears to be lowest with narrow-spectrum antibiotics (123, 124).

### 5.5 Autoimmune diseases

In people with multiple sclerosis (MS), the microbiota is characterized by elevated abundances of Firmicutes, Lachnospiraceae, *Bifidobacterium*, *Roseburia*, *Coprococcus*, *Butyricicoccus*, *Lachnospira*, *Dorea*, *Faecalibacterium*, and *Prevotella* (most of which produce SCFAs) and elevated abundances of Bacteroidetes, *Akkermansia*, *Blautia*, and *Ruminocococcus* (125). Jangri et al. used 16S rRNA sequencing and gene expression analysis to study the microbiome in 60 people with MS and 43 healthy controls. The MS group presented elevated abundances of *Methanobrevibacter* and *Akkermansia* and a lower abundance of *Butyricimonas*.

Juvenile idiopathic arthritis (JIA) is the most common rheumatic disease in children. Although the multifactorial (genetic and environmental) etiology of JIA is poorly understood, antibiotic exposure in early life has been linked to the onset of JIA (126–128). For example, Hestetun et al. studied 535,294 children born in Norway from 2004 to 2012 (129). Of these, 149,534 (27.9%) were exposed to systemic antibiotics prenatally and 236,340 (44.2%) were exposed during the first 24 months postpartum. The onset of JIA was associated with postpartum antibiotic exposure (adjusted OR [95%CI] = 1.40 [1.24–1.59]) but not prenatal antibiotic exposure. Interestingly, the association was stronger in children having received sulfonamides, trimethoprim, and broad-spectrum antibiotics (129). However, reverse causality cannot be ruled out because inflammatory joint symptoms (especially in children) may be misinterpreted as resulting from a bacterial infection.

### 5.6 Obesity

Researchers have evidenced complex interactions between the diet, the gut microbiota, inflammation, and obesity. Mechanistically, the pathways involve the microbial production of energy substrates, inflammatory effects on metabolism, and even an impact on satiety through the gut-brain axis (130). There appears to be a signature microbiotic profile for obesity: obese individuals have greater abundances of *E. coli*, Lactobacillaceae, *Escherichia*, *shigella*, and Negativicutes (131, 132). A large number of population-based studies have linked antibiotic administration to mothers during pregnancy and/or to infants in the first months of life to an elevated risk of being overweight later in childhood (133–135).

### 5.7 Allergy

Impaired or delayed maturation of the microbiota (with degraded mucus, elevated intestinal permeability, and a low proportion of SCFA-producing bacteria) during the first year postpartum may be a feature of allergic disease (136, 137). Ahmadizar et al. meta-analysis of 22 studies highlighted an association between antibiotic exposure in the first 2 years of life and the subsequent diagnosis of eczema (OR: 1.26) and allergic rhinitis (OR: 1.23) (138). However, the results of the analyzed studies were somewhat contradictory and antibiotic exposure was not linked to objective measures of atopy, such as the serum specific IgE level or prick tests positivity or weald size.

Lu et al.’s analysis of asthma trajectories in the Longitudinal Study of Australian Children found that any antibiotic exposure in the first 2 years of life increased the risk [95%CI] of early-persistent asthma by a factor of 2.3 [1.47–3.67] (*p* < 0.001) (139). In an incidence density study nested within a data collection project, Bentouhami et al. assessed 1,128 mother–child pairs in Belgium (140). Excessive systemic antibiotic use in the first year of life (defined by the researchers as more than four courses) had more than twice the incidence density ratio [95%CI] of asthma (2.18 [0.98, 4.87], *p* = 0.06), relative to all other children.

However, the relationships with allergies appear to be complex, and some studies have evidenced positive associations with antibiotic exposure. For example, Schoch et al. retrospectively studied 4,106 infants in Florida from 2011 to 2017, roughly half of whom had received antibiotics during the study period (141). Antibiotic exposure (as noted in electronic health records) during the first year of life (and especially during the first month of life) was associated with a lower risk of atopic dermatitis. The researchers suggested that there may be a “critical window” for immune tolerance in human infants, which is influenced by antibiotic exposure (141).

### 5.8 Kawasaki disease

Dysbiosis has been reported as a susceptibility factor in Kawasaki disease (KD) (142–144). Teramato used 16S rRNA gene analysis to characterize the fecal microbiota of 26 children with KD and 57 age-matched healthy controls (median age, 36.0 months). The KD group had a higher relative abundance of pro-inflammatory *Ruminococcus gnavus* and lower relative abundance of butyrate-producing *Blautia* spp. (144). Antibiotic exposure might be a factor in the physiopathology of KD. Kim et al. studied 17,818 *c*hildren aged under 5 diagnosed with KD between 2016 and 2019, together with 89,090 matched controls. Use of antibiotics in the previous 6 or 12 months was associated with the development of KD (OR [95%CI]: 1.18 [1.12–1.26] and 1.23 [1.14–1.32], respectively). The researchers suggested that antibiotic-related changes in the gut microbiota might have a role in the development of KD (145). Kim et al. findings were in line with those of a previous study in Japan (146). However, Burns has pointed out that the establishment of a causal dysbiotic relationship between antibiotic exposure and KD would require adequately powered studies with appropriate matching criteria and a comparison of fecal samples from patients with KD vs. samples from patients without KD but similar levels of inflammation (147).

### 5.9 Atherosclerosis

In a study of the atherosclerosis-prone apolipoprotein E-knockout mouse model, Kappel et al. used 16S ribosomal RNA serum metabolomics to evidence an antibiotic-induced fall in the abundance of certain Bacteroidetes and Clostridia. Antibiotic administration was associated with a greater atherosclerotic lesion size, independently of diet. The results of a serum metabolome analysis was suggestive of disturbances in tryptophan, trimethylamine-N-oxide and lipid metabolism by the gut microbiota (148).

### 5.10 Cancer

A moderate body of evidence suggests that excessive or prolonged antibiotic use is associated not only with a slightly greater risk of cancer onset but also a relative reduction in the effectiveness of cancer treatments (encompassing chemotherapy, radiotherapy, and immunotherapy) (149–151). The strength of the association between antibiotic exposure and cancer onset varies from one type of cancer to another and from one class of antibiotics to another (152). Relationships have been shown for breast cancer, endocrine gland cancers, pancreatic cancer and (to a lesser extent) lung cancer, esophageal cancer, gastric cancer, and ovarian cancer (150–155). Unsurprisingly (in view of the extreme proximity to the gut and the impact on public health), colorectal cancer has been extensively investigated with regard to antibiotic exposure. Most investigators have found a significant, dose-dependent association with colon cancer but not with rectal cancer (156, 157). The elevated risk of colon cancer might be related to low SCFA levels.

## 6 Mitigation of the potential long-term negative health effects of antibiotic exposure

The quotation “all disease begins in the gut” is often attributed to Hippocrates *circa* 400 BC. The father of modern medicine was probably not fully correct but, as seen for the diseases reviewed above, the DOHaD concept can be logically extended to what we term the “gastrointestinal origins of health and disease” (“GOHaD”) or even the “microbiotic origins of health and disease” (“MOHaD”). It should nevertheless be borne in mind that the ORs for the associations between antibiotic exposure and the onset of chronic disease are generally quite low (i.e., between 1 and 1.5) and, despite the investigators’ best efforts in study design and data analysis, may be influenced by confounding factors.

More generally, we found that most of the clinical data on the chronic effects of antibiotics were generated in Europe and in North America. Further research in low- and middle-income countries is warranted because the latter are especially burdened by antibiotic resistance problems, vulnerability to infections by antibiotic-resistant pathogens, and the corresponding effects on the gut microbiota (158).

Probiotics have long been viewed as a means of treating the acute gastrointestinal disorders associated with antibiotic-associated gut dysbiosis (8, 159–161). Can probiotics be recommended as adjunct treatments to mitigate the negative effects of antibiotics? The European Society for Paediatric Gastroenterology Hepatology and Nutrition (ESPGHAN) recommends strain-specific probiotics for the prevention of antibiotic-associated diarrhea in children, whereas the American Gastroenterological Association (AGA) guidelines indicate that strain-specific probiotics may be used to prevent *Clostridiodes difficile* infections (162, 163). For adults and children with IBDs like CD or UC, the AGA guidelines only recommend the use of probiotics only in the context of a clinical trial (162). Similarly, the guidelines issued by the European Crohn’s and Colitis Organisation and the ESPGHAN state that in patients with CD, probiotics should not be used to induce or maintain remission (163–165). According to the guidelines issued by the World Gastroenterology Organisation, there is evidence of strain-specific efficacy of probiotics in the prevention of antibiotic-associated diarrhea in adults or children who are receiving antibiotic therapy (166).

Hence, in view of the links between gut dysbiosis and the chronic diseases described above, one can reasonably hypothesize that the administration of probiotics will provide a degree of disease modification or symptom prophylaxis and mitigate the long-term consequences of antibiotic exposure. The main species to have been tested are lactic acid bacteria (such as *Lacticaseibacillus rhamnosus GG, Limosilactobacillus reuteri*, *Lacticaseibacillus paracasei, Lactiplantibacillus plantarum, Lactobacillus acidophilus, Lactobacillus helveticus*, *Bifidobacterium lactis, Bifidobacterium breve*, and *Streptococcus thermophilus*) *and the yeast Saccharomyces boulardii*. The positive reported short-term effects of probiotics in various patient populations (IBD, celiac disease, cancer, obesity, types 1 and 2 diabetes mellitus, allergic disease, idiopathic nephrotic syndrome, KD; multiple sclerosis, rheumatoid arthritis, and systemic lupus erythematosus, atherosclerosis, and neurological, neurodevelopmental and neurodegenerative diseases) will not be described in detail here because (i) the topic falls outside the scope of this review and (ii) the results have been extensively reviewed elsewhere in the literature (103, 159–161, 163–165, 167–210). Certain studies showed a positive effect of certain probiotic strains in combination with antibiotics and have provided a rationale for using probiotics to protect the gut microbiota and intestinal barrier functions.

Probiotics have been tested in animal models of chronic disease and in observational or interventional clinical studies, with moderate, variable but generally positive results: evidenced significant differences or improvements in clinical disease scores, symptom scores, and disease marker levels, whereas other studies found no benefit. In the field of cognitive and psychiatric disorders and mental health problems, the term “psychobiotic” has been used to describe probiotics that act through the gut-brain axis. Following on from extensive preclinical data on an association between antibiotic-induced gut dysbiosis and psychopathologies in the rat (211), there is preliminary evidence to suggest that specific probiotics may improve cognitive function, particularly in people with age-related mild cognitive impairment (212, 213).

In the literature on probiotics, the most common design is the randomized, controlled trial of the efficacy and safety of a probiotic in patients. The second most common design is the pharmacokinetic study, which documents the recovery and/or clearance of an oral dose of probiotic or measures pre−/post differences in the abundances of probiotic strains. According to McFarland, three models of dysbiosis have been frequently evaluated (214). In model A (restoration), probiotic therapy is initiated and studied after the microbiota of initially healthy patients has become disrupted (e.g., by antibiotic exposure). In model B (alteration), patients with a pre-existing disruption of the microbiota are studied after probiotic therapy. In model C (no dysbiosis), volunteers with no disruptive events are studied before and after probiotic therapy. In McFarland’s systematic review of 63 trials (published in 2014), 83% of the probiotic products evaluated in model A restored the microbiota. The corresponding proportion was 56% in model B. Only 21% of the probiotics evaluated in model C had an effect on the microbiota. Clinical efficacy was more commonly observed for with strains capable of restoring the normal microbiota (214).

Furthermore, meta-analyses of trials in better-studied disease areas have sometimes failed to show a clear, beneficial effect of probiotics. However, the meta-analyses’ authors almost always highlight the degree of interstudy heterogeneity with regard to probiotic doses, strains, and treatment durations. Larger, multicenter, randomized, controlled trial of probiotics (possibly simultaneously investigating the gut microbiota and disease markers) are warranted.

The gut virome and gut mycobiome have attracted less attention than the gut’s bacterial communities but, as mentioned above, are known to be abnormal in people with IBD (107–109, 215). We recommend further investigation of the potential indirect effects of antibiotic treatment and probiotics on the gut virome and mycobiome. Specific strains of yeast probiotics are a topic of interest; as mentioned above, one of the most effective and frequently evaluated probiotics is a yeast (*Saccharomyces boulardii* CNCM I-745) and therefore is not directly affected by antibiotics (8, 216–218). *Saccharomyces boulardii* is not a natural member of the human gut microbiota and is eliminated rapidly after probiotic administration is discontinued. However, when present as a probiotic, certain strains of *S. boulardii* exert several beneficial actions (including protection of the mucus layer, the stimulation of SCFA production by Lachnospiraceae and Ruminococcaceae, and a reduction in local inflammation) that counter antibiotic-associated dysbiosis (8, 216–218). In terms of the composition of the microbiota, treatment with *S. boulardii* is associated with increased abundances of Bacteroidaceae and Prevotellaceae and the suppression of pioneer bacteria (218). Treatment with *S. boulardii* CNCM I-745 can mitigate antibiotic-associated dysbiosis and diarrhea (219). More studies are needed to explore the full potential of this versatile probiotic yeast (218).

## 7 Conclusion

The results of our review indicate that antibiotic exposure is associated with a number of negative long-term (i.e., chronic) effects on health. Gut dysbiosis might be the causal link between antibiotic exposure and these chronic negative effects, although the lack of a replicable consensus definition of dysbiosis can lead to ambiguity in the interpretation of the data. Given that certain well-studied probiotics (such as *S. boulardii* CNCM I-745 and *L. rhamnosus* GG) are inexpensive, safe and effective in preventing short-term negative consequences of antibiotic exposure (including dysbiosis), there is no reason to summarily rule out potential longer-term benefits in a particular chronic disease setting or patient population. We recommend that decisions to initiate probiotic treatment should be made on a case-by-case basis after informed, evidenced-based discussion between the patient and his/her physician.

## References

[1] Hutchings MI Truman AW Wilkinson B. Antibiotics: past, present and future. Curr Opin Microbiol. (2019) 51:72–80. doi: 10.1016/j.mib.2019.10.008 31733401

[2] Centers for Disease Control and Prevention, (2019). Antibiotic use in the United States, 2018 update: progress and opportunities. Available at: https://Www.Cdc.Gov/Antibiotic-Use/Stewardship-Report/Pdf/Stewardship-Report-2018-508.Pdf (Accessed February 20th, 2024).

[3] Bara W Brun-Buisson C Coignard B Watier L. Outpatient antibiotic prescriptions in France: patients and providers characteristics and impact of the Covid-19 pandemic. Antibiotics. (2022) 11:643. doi: 10.3390/antibiotics11050643, PMID: 35625287 PMC9137595

[4] Fernandez-Urrusuno R Meseguer Barros CM Anaya-Ordonez S Borrego Izquierdo Y Lallana-Alvarez MJ Madridejos R. Patients receiving a high burden of antibiotics in the Community in Spain: a cross-sectional study. Pharmacol Res Perspect. (2021) 9:e00692. doi: 10.1002/prp2.692, PMID: 33340264 PMC7749514

[5] Fink G D'Acremont V Leslie HH Cohen J. Antibiotic exposure among children younger than 5 years in low-income and middle-income countries: a cross-sectional study of nationally representative facility-based and household-based surveys. Lancet Infect Dis. (2020) 20:179–87. doi: 10.1016/S1473-3099(19)30572-9, PMID: 31843383

[6] Sijbom M Buchner FL Saadah NH Numans ME de Boer MGJ. Determinants of inappropriate antibiotic prescription in primary care in developed countries with general practitioners as gatekeepers: a systematic review and construction of a framework. BMJ Open. (2023) 13:e065006. doi: 10.1136/bmjopen-2022-065006, PMID: 37197815 PMC10193070

[7] Kapoor G Saigal S Elongavan A. Action and resistance mechanisms of antibiotics: a guide for clinicians. J Anaesthesiol Clin Pharmacol. (2017) 33:300–5. doi: 10.4103/joacp.JOACP_349_15, PMID: 29109626 PMC5672523

[8] Waitzberg D Guarner F Hojsak I Ianiro G Polk DB Sokol H. Can the evidence-based use of probiotics (notably Saccharomyces Boulardii Cncm I-745 and *Lactobacillus rhamnosus* gg) mitigate the clinical effects of antibiotic-associated dysbiosis? Adv Ther. (2024) 41:901–14. doi: 10.1007/s12325-024-02783-3, PMID: 38286962 PMC10879266

[9] Perez NB Dorsen C Squires A. Dysbiosis of the gut microbiome: a concept analysis. J Holist Nurs. (2020) 38:223–32. doi: 10.1177/0898010119879527 31603019

[10] Zimmermann P Curtis N. The effect of antibiotics on the composition of the intestinal microbiota - a systematic review. J Infect. (2019) 79:471–89. doi: 10.1016/j.jinf.2019.10.008 31629863

[11] Palleja A Mikkelsen KH Forslund SK Kashani A Allin KH Nielsen T. Recovery of gut microbiota of healthy adults following antibiotic exposure. Nat Microbiol. (2018) 3:1255–65. doi: 10.1038/s41564-018-0257-9 30349083

[12] Patangia DV Anthony Ryan C Dempsey E Paul Ross R Stanton C. Impact of antibiotics on the human microbiome and consequences for host health. Microbiology. (2022) 11:e1260. doi: 10.1002/mbo3.1260, PMID: 35212478 PMC8756738

[13] Petersen C Round JL. Defining Dysbiosis and its influence on host immunity and disease. Cell Microbiol. (2014) 16:1024–33. doi: 10.1111/cmi.12308, PMID: 24798552 PMC4143175

[14] Uddin TM Chakraborty AJ Khusro A Zidan BRM Mitra S Emran TB. Antibiotic resistance in microbes: history, mechanisms, therapeutic strategies and future prospects. J Infect Public Health. (2021) 14:1750–66. doi: 10.1016/j.jiph.2021.10.020, PMID: 34756812

[15] Elseviers MM Van Camp Y Nayaert S Dure K Annemans L Tanghe A. Prevalence and management of antibiotic associated diarrhea in general hospitals. BMC Infect Dis. (2015) 15:129. doi: 10.1186/s12879-015-0869-0, PMID: 25888351 PMC4403881

[16] Anthony WE Wang B Sukhum KV D'Souza AW Hink T Cass C. Acute and persistent effects of commonly used antibiotics on the gut microbiome and resistome in healthy adults. Cell Rep. (2022) 39:110649. doi: 10.1016/j.celrep.2022.110649, PMID: 35417701 PMC9066705

[17] Xiang Y Li F Peng J Qin D Yuan M Liu G. Risk factors and predictive model of diarrhea among patients with severe stroke. World Neurosurg. (2020) 136:213–9. doi: 10.1016/j.wneu.2019.12.125, PMID: 31901495

[18] Zhou H Xu Q Liu Y Guo LT. Risk factors, incidence, and morbidity associated with antibiotic-associated diarrhea in intensive care unit patients receiving antibiotic monotherapy. World J Clin Cases. (2020) 8:1908–15. doi: 10.12998/wjcc.v8.i10.1908, PMID: 32518780 PMC7262719

[19] Guo Q Goldenberg JZ Humphrey C El Dib R Johnston BC. Probiotics for the prevention of pediatric antibiotic-associated diarrhea. Cochrane Database Syst Rev. (2019) 4:CD004827. doi: 10.1002/14651858.CD004827.pub5, PMID: 31039287 PMC6490796

[20] Helander HF Fandriks L. Surface area of the digestive tract - revisited. Scand J Gastroenterol. (2014) 49:681–9. doi: 10.3109/00365521.2014.898326, PMID: 24694282

[21] Marchesi JR Ravel J. The vocabulary of microbiome research: a proposal. Microbiome. (2015) 3:31. doi: 10.1186/s40168-015-0094-5, PMID: 26229597 PMC4520061

[22] Arumugam M Raes J Pelletier E Le Paslier D Yamada T Mende DR. Enterotypes of the human gut microbiome. Nature. (2011) 473:174–80. doi: 10.1038/nature09944, PMID: 21508958 PMC3728647

[23] Robertson RC Manges AR Finlay BB Prendergast AJ. The human microbiome and child growth - first 1000 days and beyond. Trends Microbiol. (2019) 27:131–47. doi: 10.1016/j.tim.2018.09.008, PMID: 30529020

[24] Romano-Keeler J Sun J. The first 1000 days: assembly of the neonatal microbiome and its impact on health outcomes. Newborn. (2022) 1:219–26. doi: 10.5005/jp-journals-11002-0028, PMID: 36237439 PMC9555117

[25] Mustard JF. Canadian Progress in early child development - putting science into action. Paediatr Child Health. (2009) 14:689–90. doi: 10.1093/pch/14.10.689 PMID: 21119821 PMC2807816

[26] Shonkoff JP Richter L van der Gaag J Bhutta ZA. An integrated scientific framework for child survival and early childhood development. Pediatrics. (2012) 129:e460–72. doi: 10.1542/peds.2011-0366, PMID: 22218840

[27] Black RE Victora CG Walker SP Bhutta ZA Christian P de Onis M. Maternal and child undernutrition and overweight in low-income and middle-income countries. Lancet. (2013) 382:427–51. doi: 10.1016/S0140-6736(13)60937-X 23746772

[28] Bryce J Coitinho D Darnton-Hill I Pelletier D Pinstrup-Andersen P. Maternal and child undernutrition: effective action at National Level. Lancet. (2008) 371:510–26. doi: 10.1016/S0140-6736(07)61694-8 18206224

[29] Beluska-Turkan K Korczak R Hartell B Moskal K Maukonen J Alexander DE. Nutritional gaps and supplementation in the first 1000 days. Nutrients. (2019) 11:2891. doi: 10.3390/nu11122891, PMID: 31783636 PMC6949907

[30] Scott JA. The first 1000 days: a critical period of nutritional opportunity and vulnerability. Nutr Diet. (2020) 77:295–7. doi: 10.1111/1747-0080.12617, PMID: 32478460

[31] Hoffman DJ Reynolds RM Hardy DB. Developmental origins of health and disease: current knowledge and potential mechanisms. Nutr Rev. (2017) 75:951–70. doi: 10.1093/nutrit/nux053, PMID: 29186623

[32] Ryznar RJ Phibbs L Van Winkle LJ. Epigenetic modifications at the Center of the Barker Hypothesis and Their Transgenerational Implications. Int J Environ Res Public Health. (2021) 18:12728. doi: 10.3390/ijerph182312728, PMID: 34886453 PMC8656758

[33] Barker DJ. The fetal and infant origins of adult disease. BMJ. (1990) 301:1111. doi: 10.1136/bmj.301.6761.1111, PMID: 2252919 PMC1664286

[34] Banchi P Colitti B Opsomer G Rota A Van Soom A. The dogma of the sterile uterus revisited: does microbial seeding occur during fetal life in humans and animals? Reproduction. (2024) 167:e230078. doi: 10.1530/REP-23-0078, PMID: 37903182 PMC10762539

[35] Aagaard KM. Author response to comment on "the placenta harbors a unique microbiome". Sci Transl Med. (2014) 6:254lr3. doi: 10.1126/scitranslmed.3010007, PMID: 25232176

[36] Collado MC Rautava S Aakko J Isolauri E Salminen S. Human gut colonisation may be initiated in utero by distinct microbial communities in the placenta and amniotic fluid. Sci Rep. (2016) 6:23129. doi: 10.1038/srep23129, PMID: 27001291 PMC4802384

[37] Kennedy KM de Goffau MC Perez-Munoz ME Arrieta MC Backhed F Bork P. Questioning the fetal microbiome illustrates pitfalls of low-biomass microbial studies. Nature. (2023) 613:639–49. doi: 10.1038/s41586-022-05546-8, PMID: 36697862 PMC11333990

[38] Perez-Munoz ME Arrieta MC Ramer-Tait AE Walter J. A critical assessment of the "sterile womb" and "in utero colonization" hypotheses: implications for research on the Pioneer infant microbiome. Microbiome. (2017) 5:48. doi: 10.1186/s40168-017-0268-4 28454555 PMC5410102

[39] Romano-Keeler J Weitkamp JH. Maternal influences on fetal microbial colonization and immune development. Pediatr Res. (2015) 77:189–95. doi: 10.1038/pr.2014.163, PMID: 25310759 PMC4289016

[40] Yassour M Vatanen T Siljander H Hamalainen AM Harkonen T Ryhanen SJ. Natural history of the infant gut microbiome and impact of antibiotic treatment on bacterial strain diversity and stability. Sci Transl Med. (2016) 8:343ra81. doi: 10.1126/scitranslmed.aad0917, PMID: 27306663 PMC5032909

[41] Fouhy F Watkins C Hill CJ O'Shea CA Nagle B Dempsey EM. Perinatal factors affect the gut microbiota up to four years after birth. Nat Commun. (2019) 10:1517. doi: 10.1038/s41467-019-09252-4, PMID: 30944304 PMC6447568

[42] Salminen S Gibson GR McCartney AL Isolauri E. Influence of mode of delivery on gut microbiota composition in seven year old children. Gut. (2004) 53:1388–9. doi: 10.1136/gut.2004.041640, PMID: 15306608 PMC1774211

[43] Cetinbas M Thai J Filatava E Gregory KE Sadreyev RI. Long-term dysbiosis and fluctuations of gut microbiome in antibiotic treated preterm infants. iScience. (2023) 26:107995. doi: 10.1016/j.isci.2023.107995 37829203 PMC10565780

[44] Parkin K Christophersen CT Verhasselt V Cooper MN Martino D. Risk factors for gut Dysbiosis in early life. Microorganisms. (2021) 9:2066. doi: 10.3390/microorganisms9102066, PMID: 34683389 PMC8541535

[45] Kwon Y Cho YS Lee YM Kim SJ Bae J Jeong SJ. Changes to gut microbiota following systemic antibiotic Administration in Infants. Antibiotics. (2022) 11:470. doi: 10.3390/antibiotics11040470, PMID: 35453221 PMC9025670

[46] Schulman J Dimand RJ Lee HC Duenas GV Bennett MV Gould JB. Neonatal intensive care unit antibiotic use. Pediatrics. (2015) 135:826–33. doi: 10.1542/peds.2014-3409 25896845

[47] Leroux S Zhao W Betremieux P Pladys P Saliba E Jacqz-Aigrain E. Therapeutic guidelines for prescribing antibiotics in neonates should be evidence-based: a French National Survey. Arch Dis Child. (2015) 100:394–8. doi: 10.1136/archdischild-2014-306873, PMID: 25628457

[48] Kadambari S Heath PT Sharland M Lewis S Nichols A Turner MA. Variation in gentamicin and vancomycin dosage and monitoring in UK neonatal units. J Antimicrob Chemother. (2011) 66:2647–50. doi: 10.1093/jac/dkr351 21862473

[49] Wilkins LJ Monga M Miller AW. Defining dysbiosis for a cluster of chronic diseases. Sci Rep. (2019) 9:12918. doi: 10.1038/s41598-019-49452-y, PMID: 31501492 PMC6733864

[50] Brussow H. Problems with the concept of gut microbiota dysbiosis. Microb Biotechnol. (2020) 13:423–34. doi: 10.1111/1751-7915.13479, PMID: 31448542 PMC7017827

[51] Drago L Valentina C Fabio P. Gut microbiota, dysbiosis and Colon lavage. Dig Liver Dis. (2019) 51:1209–13. doi: 10.1016/j.dld.2019.06.012, PMID: 31358483

[52] Bidell MR Hobbs ALV Lodise TP. Gut microbiome health and dysbiosis: a clinical primer. Pharmacotherapy. (2022) 42:849–57. doi: 10.1002/phar.2731, PMID: 36168753 PMC9827978

[53] Levy M Kolodziejczyk AA Thaiss CA Elinav E. Dysbiosis and the immune system. Nat Rev Immunol. (2017) 17:219–32. doi: 10.1038/nri.2017.7 28260787

[54] Malard F Dore J Gaugler B Mohty M. Introduction to host microbiome symbiosis in health and disease. Mucosal Immunol. (2021) 14:547–54. doi: 10.1038/s41385-020-00365-4, PMID: 33299088 PMC7724625

[55] Chriett S Dabek A Wojtala M Vidal H Balcerczyk A Pirola L. Prominent action of butyrate over beta-hydroxybutyrate as histone deacetylase inhibitor, transcriptional modulator and anti-inflammatory molecule. Sci Rep. (2019) 9:742. doi: 10.1038/s41598-018-36941-9, PMID: 30679586 PMC6346118

[56] Kaminsky LW Al-Sadi R Ma TY. Il-1beta and the intestinal epithelial tight junction barrier. Front Immunol. (2021) 12:767456. doi: 10.3389/fimmu.2021.767456, PMID: 34759934 PMC8574155

[57] De Vadder F Grasset E Manneras Holm L Karsenty G Macpherson AJ Olofsson LE. Gut microbiota regulates maturation of the adult enteric nervous system via enteric serotonin networks. Proc Natl Acad Sci USA. (2018) 115:6458–63. doi: 10.1073/pnas.1720017115, PMID: 29866843 PMC6016808

[58] Grondin JA Khan WI. Emerging roles of gut serotonin in regulation of immune response, microbiota composition and intestinal inflammation. J Can Assoc Gastroenterol. (2024) 7:88–96. doi: 10.1093/jcag/gwad020, PMID: 38314177 PMC10836984

[59] Hou Y Li J Ying S. Tryptophan metabolism and gut microbiota: a novel regulatory Axis integrating the microbiome, immunity, and cancer. Metabolites. (2023) 13:1166. doi: 10.3390/metabo13111166, PMID: 37999261 PMC10673612

[60] Wall R Cryan JF Ross RP Fitzgerald GF Dinan TG Stanton C. Bacterial neuroactive compounds produced by psychobiotics. Adv Exp Med Biol. (2014) 817:221–39. doi: 10.1007/978-1-4939-0897-4_10, PMID: 24997036

[61] Dahiya D Manuel JV Nigam PS. An overview of bioprocesses employing specifically selected microbial catalysts for gamma-aminobutyric acid production. Microorganisms. (2021) 9:2457. doi: 10.3390/microorganisms9122457, PMID: 34946060 PMC8704203

[62] Ilan Y. Leaky gut and the liver: a role for bacterial translocation in nonalcoholic steatohepatitis. World J Gastroenterol. (2012) 18:2609–18. doi: 10.3748/wjg.v18.i21.2609, PMID: 22690069 PMC3369997

[63] Mu Q Kirby J Reilly CM Luo XM. Leaky gut as a danger signal for autoimmune diseases. Front Immunol. (2017) 8:598. doi: 10.3389/fimmu.2017.00598 28588585 PMC5440529

[64] Sadagopan A Mahmoud A Begg M Tarhuni M Fotso M Gonzalez NA. Understanding the role of the gut microbiome in diabetes and therapeutics targeting leaky gut: a systematic review. Cureus. (2023) 15:e41559. doi: 10.7759/cureus.41559, PMID: 37554593 PMC10405753

[65] Kenny DJ Plichta DR Shungin D Koppel N Hall AB Fu B. Cholesterol metabolism by uncultured human gut bacteria influences host cholesterol level. Cell Host Microbe. (2020) 28:245–257.e6. doi: 10.1016/j.chom.2020.05.013, PMID: 32544460 PMC7435688

[66] Kriaa A Bourgin M Potiron A Mkaouar H Jablaoui A Gerard P. Microbial impact on cholesterol and bile acid metabolism: current status and future prospects. J Lipid Res. (2019) 60:323–32. doi: 10.1194/jlr.R088989, PMID: 30487175 PMC6358303

[67] Black CJ Drossman DA Talley NJ Ruddy J Ford AC. Functional gastrointestinal disorders: advances in understanding and management. Lancet. (2020) 396:1664–74. doi: 10.1016/S0140-6736(20)32115-2 33049221

[68] Faye AS Allin KH Iversen AT Agrawal M Faith J Colombel JF. Antibiotic use as a risk factor for inflammatory bowel disease across the ages: a population-based cohort study. Gut. (2023) 72:663–70. doi: 10.1136/gutjnl-2022-327845, PMID: 36623926 PMC9998355

[69] Kim M Park SJ Choi S Chang J Kim SM Jeong S. Association between antibiotics and dementia risk: a retrospective cohort study. Front Pharmacol. (2022) 13:888333. doi: 10.3389/fphar.2022.888333, PMID: 36225572 PMC9548656

[70] Sanchez E Donat E Ribes-Koninckx C Fernandez-Murga ML Sanz Y. Duodenal-mucosal bacteria associated with celiac disease in children. Appl Environ Microbiol. (2013) 79:5472–9. doi: 10.1128/AEM.00869-13, PMID: 23835180 PMC3754165

[71] Champagne-Jorgensen K Kunze WA Forsythe P Bienenstock J McVey Neufeld KA. Antibiotics and the nervous system: more than just the microbes? Brain Behav Immun. (2019) 77:7–15. doi: 10.1016/j.bbi.2018.12.014, PMID: 30582961

[72] Rybak LP Ramkumar V Mukherjea D. Ototoxicity of non-aminoglycoside antibiotics. Front Neurol. (2021) 12:652674. doi: 10.3389/fneur.2021.652674, PMID: 33767665 PMC7985331

[73] Fusco W Lorenzo MB Cintoni M Porcari S Rinninella E Kaitsas F. Short-chain fatty-acid-producing Bacteria: key components of the human gut microbiota. Nutrients. (2023) 15:2211. doi: 10.3390/nu15092211 37432351 PMC10180739

[74] LA Stoddart Smith NJ Milligan G. Free fatty acid receptors Ffa1, −2, and −3: pharmacology and pathophysiological functions. Pharmacol Rev. (2008) 60:405–17. doi: 10.1124/pr.108.00802, PMID: 19047536

[75] Alvarez-Curto E Milligan G. Metabolism meets immunity: the role of free fatty acid receptors in the immune system. Biochem Pharmacol. (2016) 114:3–13. doi: 10.1016/j.bcp.2016.03.017, PMID: 27002183

[76] Kotlo K Anbazhagan AN Priyamvada S Jayawardena D Kumar A Chen Y. The olfactory G protein-coupled receptor (Olfr-78/Or51e2) modulates the intestinal response to colitis. Am J Physiol Cell Physiol. (2020) 318:C502–13. doi: 10.1152/ajpcell.00454.2019 31913697 PMC7099522

[77] Pluznick J. A novel SCFA receptor, the microbiota, and blood pressure regulation. Gut Microbes. (2014) 5:202–7. doi: 10.4161/gmic.27492, PMID: 24429443 PMC4063845

[78] Hou JJ Ma AH Qin YH. Activation of the aryl hydrocarbon receptor in inflammatory bowel disease: insights from gut microbiota. Front Cell Infect Microbiol. (2023) 13:1279172. doi: 10.3389/fcimb.2023.1279172, PMID: 37942478 PMC10628454

[79] Jin UH Cheng Y Park H Davidson LA Callaway ES Chapkin RS. Short chain fatty acids enhance aryl hydrocarbon (ah) responsiveness in mouse Colonocytes and Caco-2 human Colon Cancer cells. Sci Rep. (2017) 7:10163. doi: 10.1038/s41598-017-10824-x, PMID: 28860561 PMC5579248

[80] Kespohl M Vachharajani N Luu M Harb H Pautz S Wolff S. The microbial metabolite butyrate induces expression of Th1-associated factors in Cd4(+) T cells. Front Immunol. (2017) 8:1036. doi: 10.3389/fimmu.2017.01036, PMID: 28894447 PMC5581317

[81] Zhang M Zhou Q Dorfman RG Huang X Fan T Zhang H. Butyrate inhibits Interleukin-17 and generates Tregs to ameliorate colorectal colitis in rats. BMC Gastroenterol. (2016) 16:84. doi: 10.1186/s12876-016-0500-x, PMID: 27473867 PMC4967301

[82] Gasaly N Hermoso MA Gotteland M. Butyrate and the fine-tuning of colonic homeostasis: implication for inflammatory bowel diseases. Int J Mol Sci. (2021) 22:3061. doi: 10.3390/ijms22063061, PMID: 33802759 PMC8002420

[83] Tan C Yan Q Ma Y Fang J Yang Y. Recognizing the role of the Vagus nerve in depression from microbiota-gut brain Axis. Front Neurol. (2022) 13:1015175. doi: 10.3389/fneur.2022.1015175, PMID: 36438957 PMC9685564

[84] Hung LY Boonma P Unterweger P Parathan P Haag A Luna RA. Neonatal antibiotics disrupt motility and enteric neural circuits in mouse Colon. Cell Mol Gastroenterol Hepatol. (2019) 8:298–300.e6. doi: 10.1016/j.jcmgh.2019.04.009, PMID: 31022477 PMC6717783

[85] Bernabe G Shalata MEM Zatta V Bellato M Porzionato A Castagliuolo I. Antibiotic treatment induces long-lasting effects on gut microbiota and the enteric nervous system in mice. Antibiotics (Basel). (2023) 12:1000. doi: 10.3390/antibiotics12061000, PMID: 37370319 PMC10295661

[86] Donohoe DR Garge N Zhang X Sun W O'Connell TM Bunger MK. The microbiome and butyrate regulate energy metabolism and autophagy in the mammalian Colon. Cell Metab. (2011) 13:517–26. doi: 10.1016/j.cmet.2011.02.018, PMID: 21531334 PMC3099420

[87] Ghosh SS Wang J Yannie PJ Ghosh S. Intestinal barrier dysfunction, Lps translocation, and disease development. J Endocr Soc. (2020) 4:bvz039. doi: 10.1210/jendso/bvz039, PMID: 32099951 PMC7033038

[88] Lu YC Yeh WC Ohashi PS. Lps/Tlr4 signal transduction pathway. Cytokine. (2008) 42:145–51. doi: 10.1016/j.cyto.2008.01.006 18304834

[89] Tremblay MS Aubert S Barnes JD Saunders TJ Carson V Latimer-Cheung AE. Sedentary behavior research network (SBRN) - terminology consensus project process and outcome. Int J Behav Nutr Phys Act. (2017) 14:75. doi: 10.1186/s12966-017-0525-8, PMID: 28599680 PMC5466781

[90] Carrera-Bastos P Fontes-Villalba M O’Keefe J. The Western diet and lifestyle and diseases of civilization. Res Rep Clin Cardiol. (2011) 2:15–35. doi: 10.2147/RRCC.S16919

[91] Kopp W. How Western diet and lifestyle drive the pandemic of obesity and civilization diseases. Diabetes Metab Syndr Obes. (2019) 12:2221–36. doi: 10.2147/DMSO.S216791, PMID: 31695465 PMC6817492

[92] Patterson R McNamara E Tainio M de Sa TH Smith AD Sharp SJ. Sedentary behaviour and risk of all-cause, cardiovascular and cancer mortality, and incident type 2 diabetes: a systematic review and dose response meta-analysis. Eur J Epidemiol. (2018) 33:811–29. doi: 10.1007/s10654-018-0380-1, PMID: 29589226 PMC6133005

[93] Bai J Hu Y Bruner DW. Composition of gut microbiota and its association with body mass index and lifestyle factors in a cohort of 7-18 years old children from the American gut project. Pediatr Obes. (2019) 14:e12480. doi: 10.1111/ijpo.12480, PMID: 30417607

[94] Castellanos N Diez GG Antunez-Almagro C Bailen M Bressa C Gonzalez Soltero R. A critical mutualism - competition interplay underlies the loss of microbial diversity in sedentary lifestyle. Front Microbiol. (2019) 10:3142. doi: 10.3389/fmicb.2019.03142, PMID: 32038575 PMC6987436

[95] Houttu V Boulund U Nicolaou M Holleboom AG Grefhorst A Galenkamp H. Physical activity and dietary composition relate to differences in gut microbial patterns in a multi-ethnic cohort-the Helius study. Metabolites. (2021) 11:858. doi: 10.3390/metabo11120858, PMID: 34940616 PMC8707449

[96] Tarracchini C Fontana F Lugli GA Mancabelli L Alessandri G Turroni F. Investigation of the ecological link between recurrent microbial human gut communities and physical activity. Microbiol Spectr. (2022) 10:e0042022. doi: 10.1128/spectrum.00420-22 35377222 PMC9045144

[97] Kulecka M Fraczek B Mikula M Zeber-Lubecka N Karczmarski J Paziewska A. The composition and richness of the gut microbiota differentiate the top polish endurance athletes from sedentary controls. Gut Microbes. (2020) 11:1374–84. doi: 10.1080/19490976.2020.1758009, PMID: 32401138 PMC7524299

[98] Flint HJ. The impact of nutrition on the human microbiome. Nutr Rev. (2012) 70:S10–3. doi: 10.1111/j.1753-4887.2012.00499.x 22861801

[99] Graf D Di Cagno R Fak F Flint HJ Nyman M Saarela M. Contribution of diet to the composition of the human gut microbiota. Microb Ecol Health Dis. (2015) 26:26164. doi: 10.3402/mehd.v26.26164 25656825 PMC4318938

[100] Sanchez Cruz C Rojas Huerta A Lima Barrientos J Rodriguez C Devani A Boosahda V. Inflammatory bowel disease and cardiovascular disease: an integrative review with a focus on the gut microbiome. Cureus. (2024) 16:e65136. doi: 10.7759/cureus.65136, PMID: 39170992 PMC11338650

[101] Yang Z Yang M Deehan EC Cai C Madsen KL Wine E. Dietary Fiber for the prevention of childhood obesity: a focus on the involvement of the gut microbiota. Gut Microbes. (2024) 16:2387796. doi: 10.1080/19490976.2024.2387796, PMID: 39163556 PMC11340751

[102] Garcia K Ferreira G Reis F Viana SI. Impact of dietary sugars on gut microbiota and metabolic health. Diabetology. (2022) 3:549–60. doi: 10.3390/diabetology3040042

[103] Do MH Lee E Oh MJ Kim Y Park HY. High-glucose or -fructose diet cause changes of the gut microbiota and metabolic disorders in mice without body weight change. Nutrients. (2018) 10:761. doi: 10.3390/nu10060761, PMID: 29899272 PMC6024874

[104] Laffin M Fedorak R Zalasky A Park H Gill A Agrawal A. A high-sugar diet rapidly enhances susceptibility to colitis via depletion of luminal short-chain fatty acids in mice. Sci Rep. (2019) 9:12294. doi: 10.1038/s41598-019-48749-2, PMID: 31444382 PMC6707253

[105] Aversa Z Atkinson EJ Schafer MJ Theiler RN Rocca WA Blaser MJ. Association of Infant Antibiotic Exposure with childhood health outcomes. Mayo Clin Proc. (2021) 96:66–77. doi: 10.1016/j.mayocp.2020.07.019, PMID: 33208243 PMC7796951

[106] Hassouneh SA Loftus M Yooseph S. Linking inflammatory bowel disease symptoms to changes in the gut microbiome structure and function. Front Microbiol. (2021) 12:673632. doi: 10.3389/fmicb.2021.673632, PMID: 34349736 PMC8326577

[107] Andoh A Nishida A. Alteration of the gut microbiome in inflammatory bowel disease. Digestion. (2023) 104:16–23. doi: 10.1159/000525925 35901721

[108] Imai T Inoue R Kawada Y Morita Y Inatomi O Nishida A. Characterization of fungal Dysbiosis in Japanese patients with inflammatory bowel disease. J Gastroenterol. (2019) 54:149–59. doi: 10.1007/s00535-018-1530-7, PMID: 30478724

[109] Sartor RB Wu GD. Roles for intestinal Bacteria, viruses, and Fungi in pathogenesis of inflammatory bowel diseases and therapeutic approaches. Gastroenterology. (2017) 152:327–339.e4. doi: 10.1053/j.gastro.2016.10.012, PMID: 27769810 PMC5511756

[110] Beaugerie L Langholz E Nyboe-Andersen N Pigneur B Sokol H Epicom E. Differences in epidemiological features between ulcerative colitis and Crohn's disease: the early life-programmed versus late dysbiosis hypothesis. Med Hypotheses. (2018) 115:19–21. doi: 10.1016/j.mehy.2018.03.009, PMID: 29685190

[111] Ungaro R Bernstein CN Gearry R Hviid A Kolho KL Kronman MP. Antibiotics associated with increased risk of new-onset Crohn's disease but not ulcerative colitis: a meta-analysis. Am J Gastroenterol. (2014) 109:1728–38. doi: 10.1038/ajg.2014.246, PMID: 25223575

[112] Troelsen FS Jick S. Antibiotic use in childhood and adolescence and risk of inflammatory bowel disease: a case-control study in the UK clinical practice research datalink. Inflamm Bowel Dis. (2020) 26:440–7. doi: 10.1093/ibd/izz137, PMID: 31265060

[113] Holota Y Dovbynchuk T Kaji I Vareniuk I Dzyubenko N Chervinska T. The long-term consequences of antibiotic therapy: role of colonic short-chain fatty acids (SCFA) system and intestinal barrier integrity. PLoS One. (2019) 14:e0220642. doi: 10.1371/journal.pone.0220642, PMID: 31437166 PMC6705842

[114] Saffouri GB Shields-Cutler RR Chen J Yang Y Lekatz HR Hale VL. Small intestinal microbial dysbiosis underlies symptoms associated with functional gastrointestinal disorders. Nat Commun. (2019) 10:2012. doi: 10.1038/s41467-019-09964-7, PMID: 31043597 PMC6494866

[115] Jones MP Shah A Walker MM Koloski NA Holtmann G Talley NJ. Antibiotic use but not gastrointestinal infection frequently precedes first diagnosis of functional gastrointestinal disorders. United European Gastroenterol J. (2021) 9:1074–80. doi: 10.1002/ueg2.12164, PMID: 34653313 PMC8598965

[116] Dydensborg Sander S Nybo Andersen AM Murray JA Karlstad O Husby S Stordal K. Association between antibiotics in the first year of life and celiac disease. Gastroenterology. (2019) 156:2217–29. doi: 10.1053/j.gastro.2019.02.039, PMID: 30836095

[117] Sanz Y. Effects of a gluten-free diet on gut microbiota and immune function in healthy adult humans. Gut Microbes. (2010) 1:135–7. doi: 10.4161/gmic.1.3.11868, PMID: 21327021 PMC3023594

[118] Dash S Syed YA Khan MR. Understanding the role of the gut microbiome in brain development and its association with neurodevelopmental psychiatric disorders. Front Cell Dev Biol. (2022) 10:880544. doi: 10.3389/fcell.2022.880544, PMID: 35493075 PMC9048050

[119] Lacorte E Gervasi G Bacigalupo I Vanacore N Raucci U Parisi P. A systematic review of the microbiome in children with neurodevelopmental disorders. Front Neurol. (2019) 10:727. doi: 10.3389/fneur.2019.00727, PMID: 31417479 PMC6682593

[120] Lewandowska-Pietruszka Z Figlerowicz M Mazur-Melewska K. Microbiota in autism Spectrum disorder: a systematic review. Int J Mol Sci. (2023) 24:16660. doi: 10.3390/ijms242316660, PMID: 38068995 PMC10706819

[121] Tao Q Shen Y Li Y Luo H Yuan M Gan J. Prenatal exposure to antibiotics and risk of neurodevelopmental disorders in offspring: a systematic review and meta-analysis. Front Neurol. (2022) 13:1045865. doi: 10.3389/fneur.2022.1045865 36504646 PMC9732381

[122] Slykerman RF Thompson J Waldie KE Murphy R Wall C Mitchell EA. Antibiotics in the first year of life and subsequent neurocognitive outcomes. Acta Paediatr. (2017) 106:87–94. doi: 10.1111/apa.13613, PMID: 27701771

[123] Kohler O Petersen L Mors O Mortensen PB Yolken RH Gasse C. Infections and exposure to anti-infective agents and the risk of severe mental disorders: a Nationwide study. Acta Psychiatr Scand. (2017) 135:97–105. doi: 10.1111/acps.12671, PMID: 27870529

[124] Kohler-Forsberg O Petersen L Gasse C Mortensen PB Dalsgaard S Yolken RH. A Nationwide study in Denmark of the association between treated infections and the subsequent risk of treated mental disorders in children and adolescents. JAMA Psychiatry. (2019) 76:271–9. doi: 10.1001/jamapsychiatry.2018.3428 30516814 PMC6439826

[125] Ordonez-Rodriguez A Roman P Rueda-Ruzafa L Campos-Rios A Cardona D. Changes in gut microbiota and multiple sclerosis: a systematic review. Int J Environ Res Public Health. (2023) 20:4624. doi: 10.3390/ijerph20054624, PMID: 36901634 PMC10001679

[126] Arvonen M Virta LJ Pokka T Kroger L Vahasalo P. Repeated exposure to antibiotics in infancy: a predisposing factor for juvenile idiopathic arthritis or a sign of this Group's greater susceptibility to infections? J Rheumatol. (2015) 42:521–6. doi: 10.3899/jrheum.140348, PMID: 25320218

[127] Horton DB Scott FI Haynes K Putt ME Rose CD Lewis JD. Antibiotic exposure and juvenile idiopathic arthritis: a case-control study. Pediatrics. (2015) 136:e333–43. doi: 10.1542/peds.2015-0036 26195533 PMC4516942

[128] McDonnell L Gilkes A Ashworth M Rowland V Harries TH Armstrong D. Association between antibiotics and gut microbiome dysbiosis in children: systematic review and Meta-analysis. Gut Microbes. (2021) 13:1–18. doi: 10.1080/19490976.2020.1870402, PMID: 33651651 PMC7928022

[129] Hestetun S Andersen S Sanner H Stordal K. Antibiotic exposure in prenatal and early life and risk of juvenile idiopathic arthritis: a Nationwide register-based cohort study. RMD Open. (2023) 9:e003333. doi: 10.1136/rmdopen-2023-003333, PMID: 37648397 PMC10471866

[130] Asadi A Shadab Mehr N Mohamadi MH Shokri F Heidary M Sadeghifard N. Obesity and gut-microbiota-brain Axis: a narrative review. J Clin Lab Anal. (2022) 36:e24420. doi: 10.1002/jcla.24420 35421277 PMC9102524

[131] Hu J Guo P Mao R Ren Z Wen J Yang Q. Gut microbiota signature of obese adults across different classifications. Diabetes Metab Syndr Obes. (2022) 15:3933–47. doi: 10.2147/DMSO.S387523, PMID: 36601354 PMC9807070

[132] Peters BA Shapiro JA Church TR Miller G Trinh-Shevrin C Yuen E. A taxonomic signature of obesity in a large study of American adults. Sci Rep. (2018) 8:9749. doi: 10.1038/s41598-018-28126-1, PMID: 29950689 PMC6021409

[133] Ajslev TA Andersen CS Gamborg M Sorensen TI Jess T. Childhood overweight after establishment of the gut microbiota: the role of delivery mode, pre-pregnancy weight and early Administration of Antibiotics. Int J Obes. (2011) 35:522–9. doi: 10.1038/ijo.2011.27, PMID: 21386800

[134] Mueller NT Whyatt R Hoepner L Oberfield S Dominguez-Bello MG Widen EM. Prenatal exposure to antibiotics, cesarean section and risk of childhood obesity. Int J Obes. (2015) 39:665–70. doi: 10.1038/ijo.2014.180, PMID: 25298276 PMC4390478

[135] Saari A Virta LJ Sankilampi U Dunkel L Saxen H. Antibiotic exposure in infancy and risk of being overweight in the first 24 months of life. Pediatrics. (2015) 135:617–26. doi: 10.1542/peds.2014-3407, PMID: 25825533

[136] Akagawa S Kaneko K. Gut microbiota and allergic diseases in children. Allergol Int. (2022) 71:301–9. doi: 10.1016/j.alit.2022.02.004 35314107

[137] Huang H Jiang J Wang X Jiang K Cao H. Exposure to prescribed medication in early life and impacts on gut microbiota and disease development. EClinicalMedicine. (2024) 68:102428. doi: 10.1016/j.eclinm.2024.102428, PMID: 38312240 PMC10835216

[138] Ahmadizar F Vijverberg SJH Arets HGM de Boer A Lang JE Garssen J. Early-life antibiotic exposure increases the risk of developing allergic symptoms later in life: a meta-analysis. Allergy. (2018) 73:971–86. doi: 10.1111/all.13332 29105784

[139] Lu Y Wang Y Wang J Lowe AJ Grzeskowiak LE Hu YJ. Early-life antibiotic exposure and childhood asthma trajectories: a National Population-Based Birth Cohort. Antibiotics. (2023) 12:314. doi: 10.3390/antibiotics12020314, PMID: 36830225 PMC9952656

[140] Bentouhami H Bungwa MK Casas L Coenen S Weyler J. Asthma occurrence in children and early life systemic antibiotic use: an incidence density study. Allergy Asthma Clin Immunol. (2023) 19:18. doi: 10.1186/s13223-023-00773-8, PMID: 36879341 PMC9987135

[141] Schoch JJ Satcher KG Garvan CW Monir RL Neu J Lemas DJ. Association between early life antibiotic exposure and development of early childhood atopic dermatitis. JAAD Int. (2023) 10:68–74. doi: 10.1016/j.jdin.2022.11.002 36688099 PMC9850168

[142] Chen J Yue Y Wang L Deng Z Yuan Y Zhao M. Altered gut microbiota correlated with systemic inflammation in children with Kawasaki disease. Sci Rep. (2020) 10:14525. doi: 10.1038/s41598-020-71371-6, PMID: 32884012 PMC7471315

[143] Kaneko K Akagawa S Akagawa Y Kimata T Tsuji S. Our evolving understanding of Kawasaki disease pathogenesis: role of the gut microbiota. Front Immunol. (2020) 11:1616. doi: 10.3389/fimmu.2020.01616, PMID: 32793240 PMC7393004

[144] Teramoto Y Akagawa S Hori SI Tsuji S Higasa K Kaneko K. Dysbiosis of the gut microbiota as a susceptibility factor for Kawasaki disease. Front Immunol. (2023) 14:1268453. doi: 10.3389/fimmu.2023.1268453, PMID: 38022552 PMC10644744

[145] Kim TH Shin JS Kim SY Kim J. Association of previous antibiotics use and Kawasaki disease: a cohort study of 106,908 patients. Pediatr Infect Dis J. (2024) 43:643–50. doi: 10.1097/INF.0000000000004335, PMID: 38534913

[146] Fukazawa M Jr Fukazawa M Nanishi E Nishio H Ichihara K Ohga S. Previous Antibiotic Use and the development of Kawasaki disease: a matched pair case-control study. Pediatr Int. (2020) 62, 62:1044–8. doi: 10.1111/ped.14255 32306442

[147] Burns JC. The etiologies of Kawasaki disease. J Clin Invest. (2024) 134:e176938. doi: 10.1172/JCI176938 38426498 PMC10904046

[148] Kappel BA De Angelis L Heiser M Ballanti M Stoehr R Goettsch C. Cross-omics analysis revealed gut microbiome-related metabolic pathways underlying atherosclerosis development after antibiotics treatment. Mol Metab. (2020) 36:100976. doi: 10.1016/j.molmet.2020.100976, PMID: 32251665 PMC7183232

[149] Amadei SS Notario V. A significant question in cancer risk and therapy: are antibiotics positive or negative effectors? Current answers and possible alternatives. Antibiotics. (2020) 9:580. doi: 10.3390/antibiotics9090580, PMID: 32899961 PMC7558931

[150] Kilkkinen A Rissanen H Klaukka T Pukkala E Heliovaara M Huovinen P. Antibiotic use predicts an increased risk of cancer. Int J Cancer. (2008) 123:2152–5. doi: 10.1002/ijc.23622 18704945

[151] Petrelli F Ghidini M Ghidini A Perego G Cabiddu M Khakoo S. Use of antibiotics and risk of Cancer: a systematic review and Meta-analysis of observational studies. Cancers (Basel). (2019) 11:1174. doi: 10.3390/cancers11081174, PMID: 31416208 PMC6721461

[152] Cheung KS Chan EW Tam A Wong IOL Seto WK Hung IFN. Association between antibiotic consumption and colon and rectal cancer development in older individuals: a territory-wide study. Cancer Med. (2022) 11:3863–72. doi: 10.1002/cam4.4759, PMID: 35488387 PMC9582694

[153] Kim M Park SJ Choi S Jeong S Chang J Park YJ. Association of antibiotic use with risk of lung cancer: a Nationwide cohort study. J Infect Public Health. (2023) 16:1123–30. doi: 10.1016/j.jiph.2023.05.006, PMID: 37224622

[154] Tamim HM Hanley JA Hajeer AH Boivin JF Collet JP. Risk of breast cancer in relation to antibiotic use. Pharmacoepidemiol Drug Saf. (2008) 17:144–50. doi: 10.1002/pds.1512 17943999

[155] Zhang L Han DM. An introduction of allergic rhinitis and its impact on asthma (Aria) 2008 update. Zhonghua Er Bi Yan Hou Tou Jing Wai Ke Za Zhi. (2008) 43:552–7. PMID: 18826133

[156] Qu G Sun C Sharma M Uy JP Song EJ Bhan C. Is antibiotics use really associated with increased risk of colorectal cancer? An updated systematic review and meta-analysis of observational studies. Int J Color Dis. (2020) 35:1397–412. doi: 10.1007/s00384-020-03658-z, PMID: 32504337

[157] Zhang J Haines C Watson AJM Hart AR Platt MJ Pardoll DM. Oral antibiotic use and risk of colorectal cancer in the United Kingdom, 1989-2012: a matched case-control study. Gut. (2019) 68:1971–8. doi: 10.1136/gutjnl-2019-318593 31427405

[158] Luchen CC Chibuye M Spijker R Simuyandi M Chisenga C Bosomprah S. Impact of antibiotics on gut microbiome composition and Resistome in the first years of life in low- to middle-income countries: a systematic review. PLoS Med. (2023) 20:e1004235. doi: 10.1371/journal.pmed.1004235, PMID: 37368871 PMC10298773

[159] Agamennone V Krul CAM Rijkers G Kort R. A practical guide for probiotics applied to the case of antibiotic-associated diarrhea in the Netherlands. BMC Gastroenterol. (2018) 18:103. doi: 10.1186/s12876-018-0831-x, PMID: 30078376 PMC6091175

[160] Goodman C Keating G Georgousopoulou E Hespe C Levett K. Probiotics for the prevention of antibiotic-associated Diarrhoea: a systematic review and meta-analysis. BMJ Open. (2021) 11:e043054. doi: 10.1136/bmjopen-2020-043054, PMID: 34385227 PMC8362734

[161] Kopacz K Phadtare S. Probiotics for the prevention of antibiotic-associated diarrhea. Healthcare. (2022) 10:1450. doi: 10.3390/healthcare10081450, PMID: 36011108 PMC9408191

[162] Su GL Ko CW Bercik P Falck-Ytter Y Sultan S Weizman AV. Aga clinical practice guidelines on the role of probiotics in the management of gastrointestinal disorders. Gastroenterology. (2020) 159:697–705. doi: 10.1053/j.gastro.2020.05.059, PMID: 32531291

[163] Szajewska H Berni Canani R Domellof M Guarino A Hojsak I Indrio F. Probiotics for the Management of Pediatric Gastrointestinal Disorders: Position Paper of the Espghan Special Interest Group on Gut Microbiota and Modifications. J Pediatr Gastroenterol Nutr. (2023) 76:20221011. doi: 10.1097/MPG.0000000000003633 36219218

[164] Limketkai BN Akobeng AK Gordon M Adepoju AA. Probiotics for induction of remission in Crohn's disease. Cochrane Database Syst Rev. (2020) 7:CD006634. doi: 10.1002/14651858.CD006634.pub3, PMID: 32678465 PMC7389339

[165] Rolfe VE Fortun PJ Hawkey CJ Bath-Hextall F. Probiotics for maintenance of remission in Crohn's disease. Cochrane Database Syst Rev. (2006) 4:CD004826. doi: 10.1002/14651858.CD004826.pub2 17054217

[166] World Gastroenterology Organisation. Global guidelines probiotics and prebiotics. (2023). Available at: https://www.worldgastroenterology.org/userfiles/file/guidelines/probiotics-and-prebiotics-english-2023.pdf (Accessed 1st May, 2024). 10.1097/MCG.0000000000002002 38885083

[167] Andreasen AS Larsen N Pedersen-Skovsgaard T Berg RM Moller K Svendsen KD. Effects of *Lactobacillus acidophilus* NCFM on insulin sensitivity and the systemic inflammatory response in human subjects. Br J Nutr. (2010) 104:1831–8. doi: 10.1017/S0007114510002874, PMID: 20815975

[168] Ayesha IE Monson NR Klair N Patel U Saxena A Patel D. Probiotics and their role in the Management of Type 2 diabetes mellitus (short-term versus long-term effect): a systematic review and Meta-analysis. Cureus. (2023) 15:e46741. doi: 10.7759/cureus.46741, PMID: 38022046 PMC10631563

[169] Azad MB Coneys JG Kozyrskyj AL Field CJ Ramsey CD Becker AB. Probiotic supplementation during pregnancy or infancy for the prevention of asthma and wheeze: systematic review and meta-analysis. BMJ. (2013) 347:f6471. doi: 10.1136/bmj.f6471 24304677 PMC3898421

[170] Chen AC Fang TJ Ho HH Chen JF Kuo YW Huang YY. A multi-strain probiotic blend reshaped obesity-related gut Dysbiosis and improved lipid metabolism in obese children. Front Nutr. (2022) 9:922993. doi: 10.3389/fnut.2022.922993, PMID: 35990345 PMC9386160

[171] Chudzik A Orzylowska A Rola R Stanisz GJ. Probiotics, prebiotics and Postbiotics on mitigation of depression symptoms: modulation of the brain-gut-microbiome Axis. Biomol Ther. (2021) 11:1000. doi: 10.3390/biom11071000, PMID: 34356624 PMC8301955

[172] Cuello-Garcia CA Brozek JL Fiocchi A Pawankar R Yepes-Nunez JJ Terracciano L. Probiotics for the prevention of allergy: a systematic review and meta-analysis of randomized controlled trials. J Allergy Clin Immunol. (2015) 136:952–61. doi: 10.1016/j.jaci.2015.04.031, PMID: 26044853

[173] de Oliveira GLV Leite AZ Higuchi BS Gonzaga MI Mariano VS. Intestinal dysbiosis and probiotic applications in autoimmune diseases. Immunology. (2017) 152:1–12. doi: 10.1111/imm.12765, PMID: 28556916 PMC5543467

[174] de Roos NM van Hemert S Rovers JMP Smits MG Witteman BJM. The effects of a multispecies probiotic on migraine and markers of intestinal permeability-results of a randomized placebo-controlled study. Eur J Clin Nutr. (2017) 71:1455–62. doi: 10.1038/ejcn.2017.57, PMID: 28537581

[175] Di Costanzo M Vella A Infantino C Morini R Bruni S Esposito S. Probiotics in infancy and childhood for food allergy prevention and treatment. Nutrients. (2024) 16:297. doi: 10.3390/nu16020297, PMID: 38257190 PMC10819136

[176] Dosh L Ghazi M Haddad K El Masri J Hawi J Leone A. Probiotics, gut microbiome, and cardiovascular diseases: an update. Transpl Immunol. (2024) 83:102000. doi: 10.1016/j.trim.2024.102000, PMID: 38262540

[177] Fedorak RN Feagan BG Hotte N Leddin D Dieleman LA Petrunia DM. The probiotic Vsl#3 has anti-inflammatory effects and could reduce endoscopic recurrence after surgery for Crohn's disease. Clin Gastroenterol Hepatol. (2015) 13:928–935.e2. doi: 10.1016/j.cgh.2014.10.031 25460016

[178] Gao J Zhao L Cheng Y Lei W Wang Y Liu X. Probiotics for the treatment of depression and its comorbidities: a systemic review. Front Cell Infect Microbiol. (2023) 13:1167116. doi: 10.3389/fcimb.2023.1167116, PMID: 37139495 PMC10149938

[179] Garcia Vilela E de Lourdes de Abreu Ferrari M Oswaldo da Gama Torres H Guerra Pinto A Carolina Carneiro Aguirre A Paiva Martins F. Influence of Saccharomyces Boulardii on the intestinal permeability of patients with Crohn's disease in remission. Scand J Gastroenterol. (2008) 43:842–8. doi: 10.1080/00365520801943354, PMID: 18584523

[180] Guedes MR Pontes K Barreto Silva MI Neves MF Klein M. Randomized controlled trials reporting the effects of probiotics in individuals with overweight and obesity: a critical review of the interventions and body adiposity parameters. Clin Nutr. (2023) 42:835–47. doi: 10.1016/j.clnu.2023.03.017, PMID: 37084470

[181] Guo L Xu J Du Y Wu W Nie W Zhang D. Effects of gut microbiota and probiotics on Alzheimer's disease. Transl Neurosci. (2021) 12:573–80. doi: 10.1515/tnsci-2020-0203, PMID: 35070441 PMC8713066

[182] Johnson D Letchumanan V Thum CC Thurairajasingam S Lee LH. A microbial-based approach to mental health: the potential of probiotics in the treatment of depression. Nutrients. (2023) 15:1382. doi: 10.3390/nu15061382, PMID: 36986112 PMC10053794

[183] Kamarli Altun H Akal Yildiz E Akin M. Effects of Synbiotic therapy in mild-to-moderately active ulcerative colitis: a randomized placebo-controlled study. Turk J Gastroenterol. (2019) 30:313–20. doi: 10.5152/tjg.2019.18356, PMID: 30666969 PMC6453648

[184] Lau E Neves JS Ferreira-Magalhaes M Carvalho D Freitas P. Probiotic ingestion, obesity, and metabolic-related disorders: results from NHANES, 1999-2014. Nutrients. (2019) 11:1482. doi: 10.3390/nu11071482, PMID: 31261830 PMC6683043

[185] Leblhuber F Steiner K Schuetz B Fuchs D Gostner JM. Probiotic supplementation in patients with Alzheimer's dementia - an explorative intervention study. Curr Alzheimer Res. (2018) 15:1106–13. doi: 10.2174/1389200219666180813144834, PMID: 30101706 PMC6340155

[186] Leon Aguilera XE Manzano A Pirela D Bermudez V. Probiotics and gut microbiota in obesity: myths and realities of a new health revolution. J Pers Med. (2022) 12:1282. doi: 10.3390/jpm12081282, PMID: 36013231 PMC9410237

[187] Loke P Orsini F Lozinsky AC Gold M O'Sullivan MD Quinn P. Probiotic Peanut Oral immunotherapy versus Oral immunotherapy and placebo in children with Peanut allergy in Australia (Ppoit-003): a multicentre, randomised, phase 2b trial. Lancet Child Adolesc Health. (2022) 6:171–84. doi: 10.1016/S2352-4642(22)00006-2, PMID: 35123664

[188] Maftei NM Raileanu CR Balta AA Ambrose L Boev M Marin DB. The potential impact of probiotics on human health: an update on their health-promoting properties. Microorganisms. (2024) 12:234. doi: 10.3390/microorganisms12020234, PMID: 38399637 PMC10891645

[189] Makrgeorgou A Leonardi-Bee J Bath-Hextall FJ Murrell DF Tang ML Roberts A. Probiotics for treating eczema. Cochrane Database Syst Rev. (2018) 11:CD006135. doi: 10.1002/14651858.CD006135.pub3, PMID: 30480774 PMC6517242

[190] Martami F Togha M Seifishahpar M Ghorbani Z Ansari H Karimi T. The effects of a multispecies probiotic supplement on inflammatory markers and episodic and chronic migraine characteristics: a randomized double-blind controlled trial. Cephalalgia. (2019) 39:841–53. doi: 10.1177/0333102418820102, PMID: 30621517

[191] Matsuoka K Uemura Y Kanai T Kunisaki R Suzuki Y Yokoyama K. Efficacy of *Bifidobacterium breve* fermented Milk in maintaining remission of ulcerative colitis. Dig Dis Sci. (2018) 63:1910–9. doi: 10.1007/s10620-018-4946-2, PMID: 29450747 PMC6015104

[192] Meng HYH Mak CCH Mak WY Zuo T Ko H Chan FKL. Probiotic supplementation demonstrates therapeutic potential in treating gut dysbiosis and improving neurocognitive function in age-related dementia. Eur J Nutr. (2022) 61:1701–34. doi: 10.1007/s00394-021-02760-4, PMID: 35001217

[193] Merkouris E Mavroudi T Miliotas D Tsiptsios D Serdari A Christidi F. Probiotics' effects in the treatment of anxiety and depression: a comprehensive review of 2014-2023 clinical trials. Microorganisms. (2024) 12:411. doi: 10.3390/microorganisms12020411, PMID: 38399815 PMC10893170

[194] Miele E Pascarella F Giannetti E Quaglietta L Baldassano RN Staiano A. Effect of a probiotic preparation (Vsl#3) on induction and maintenance of remission in children with ulcerative colitis. Am J Gastroenterol. (2009) 104:437–43. doi: 10.1038/ajg.2008.118 19174792

[195] Mishra V Yadav D Solanki KS Koul B Song M. A review on the protective effects of probiotics against Alzheimer's disease. Biology. (2023) 13:8. doi: 10.3390/biology13010008, PMID: 38248439 PMC10813289

[196] Mozafarybazargany M Khonsari M Sokoty L Ejtahed HS Qorbani M. The effects of probiotics on gastrointestinal symptoms and microbiota in patients with celiac disease: a systematic review and meta-analysis on clinical trials. Clin Exp Med. (2023) 23:2773–88. doi: 10.1007/s10238-022-00987-x, PMID: 36609792

[197] Naghibi MM Day R Stone S Harper A. Probiotics for the prophylaxis of migraine: a systematic review of randomized placebo controlled trials. J Clin Med. (2019) 8:1441. doi: 10.3390/jcm8091441, PMID: 31514352 PMC6780403

[198] Oliva S Di Nardo G Ferrari F Mallardo S Rossi P Patrizi G. Randomised clinical trial: the effectiveness of *Lactobacillus reuteri* Atcc 55730 rectal Enema in children with active distal ulcerative colitis. Aliment Pharmacol Ther. (2012) 35:327–34. doi: 10.1111/j.1365-2036.2011.04939.x 22150569

[199] Paquette S Thomas SC Venkataraman K Appanna VD Tharmalingam S. The effects of Oral probiotics on type 2 diabetes mellitus (T2dm): a clinical trial systematic literature review. Nutrients. (2023) 15:4690. doi: 10.3390/nu15214690, PMID: 37960343 PMC10648673

[200] Paul AK Paul A Jahan R Jannat K Bondhon TA Hasan A. Probiotics and amelioration of rheumatoid arthritis: significant roles of Lactobacillus Casei and *Lactobacillus acidophilus*. Microorganisms. (2021) 9:1070. doi: 10.3390/microorganisms9051070 34065638 PMC8157104

[201] Ribeiro JF Pedrosa C. Probiotics, prebiotics and food allergy. Eur Ann Allergy Clin Immunol. (2023). doi: 10.23822/EurAnnACI.1764-1489.319 38054607

[202] Ribera C Sanchez-Orti JV Clarke G Marx W Morkl S Balanza-Martinez V. Probiotic, prebiotic, Synbiotic and fermented food supplementation in psychiatric disorders: a systematic review of clinical trials. Neurosci Biobehav Rev. (2024) 158:105561. doi: 10.1016/j.neubiorev.2024.105561, PMID: 38280441

[203] Sivamaruthi BS Kesika P Chaiyasut C. The role of probiotics in colorectal Cancer management. Evid Based Complement Alternat Med. (2020) 2020:3535982. doi: 10.1155/2020/3535982 32148539 PMC7048916

[204] Suzuki T. Regulation of the intestinal barrier by nutrients: the role of tight junctions. Anim Sci J. (2020) 91:e13357. doi: 10.1111/asj.13357, PMID: 32219956 PMC7187240

[205] Thakkar A Vora A Kaur G Akhtar J. Dysbiosis and Alzheimer's disease: role of probiotics, prebiotics and synbiotics. Naunyn Schmiedeberg's Arch Pharmacol. (2023) 396:2911–23. doi: 10.1007/s00210-023-02554-x, PMID: 37284896

[206] Yamaguchi T Tsuji S Akagawa S Akagawa Y Kino J Yamanouchi S. Clinical significance of probiotics for children with idiopathic nephrotic syndrome. Nutrients. (2021) 13:365. doi: 10.3390/nu13020365 33530312 PMC7911438

[207] Zhai T Wang P Hu X Zheng L. Probiotics bring new Hope for atherosclerosis prevention and treatment. Oxidative Med Cell Longev. (2022) 2022:3900835–13. doi: 10.1155/2022/3900835, PMID: 36193065 PMC9526629

[208] Zhang GQ Hu HJ Liu CY Zhang Q Shakya S Li ZY. Probiotics for prevention of atopy and food hypersensitivity in early childhood: a Prisma-compliant systematic review and meta-analysis of randomized controlled trials. Medicine. (2016) 95:e2562. doi: 10.1097/MD.0000000000002562, PMID: 26937896 PMC4778993

[209] Zhang Q Chen B Zhang J Dong J Ma J Zhang Y. Effect of prebiotics, probiotics, synbiotics on depression: results from a meta-analysis. BMC Psychiatry. (2023) 23:477. doi: 10.1186/s12888-023-04963-x, PMID: 37386630 PMC10308754

[210] Zhao J Liao Y Wei C Ma Y Wang F Chen Y. Potential ability of probiotics in the prevention and treatment of colorectal cancer. Clin Med Insights Oncol. (2023) 17:11795549231188225. doi: 10.1177/11795549231188225, PMID: 37601319 PMC10437046

[211] Hayer SS Hwang S Clayton JB. Antibiotic-induced gut dysbiosis and cognitive, emotional, and behavioral changes in rodents: a systematic review and meta-analysis. Front Neurosci. (2023) 17:1237177. doi: 10.3389/fnins.2023.1237177, PMID: 37719161 PMC10504664

[212] Dhyani P Goyal C Dhull SB Chauhan AK Singh Saharan B Harshita. Psychobiotics for mitigation of neuro-degenerative diseases: recent advancements. Mol Nutr Food Res. (2023):e2300461. doi: 10.1002/mnfr.202300461 37715243

[213] Ross K. Psychobiotics: are they the future intervention for managing depression and anxiety? A literature review. Explore. (2023) 19:669–80. doi: 10.1016/j.explore.2023.02.007, PMID: 36868988 PMC9940471

[214] McFarland LV. Use of probiotics to correct Dysbiosis of Normal microbiota following disease or disruptive events: a systematic review. BMJ Open. (2014) 4:e005047. doi: 10.1136/bmjopen-2014-005047 PMC4156804 25157183

[215] Liu T Asif IM Chen Y Zhang M Li B Wang L. The relationship between diet, gut Mycobiome, and functional gastrointestinal disorders: evidence, doubts, and prospects. Mol Nutr Food Res. (2024) 68:e2300382. doi: 10.1002/mnfr.202300382, PMID: 38659179

[216] Bin Z Ya-Zheng X Zhao-Hui D Bo C Li-Rong J Vandenplas Y. The efficacy of Saccharomyces Boulardii Cncm I-745 in addition to standard *Helicobacter pylori* eradication treatment in children. Pediatr Gastroenterol Hepatol Nutr. (2015) 18:17–22. doi: 10.5223/pghn.2015.18.1.17, PMID: 25866729 PMC4391996

[217] Kazmierczak-Siedlecka K Ruszkowski J Fic M Folwarski M Makarewicz W. Saccharomyces Boulardii Cncm I-745: a non-bacterial microorganism used as probiotic agent in supporting treatment of selected diseases. Curr Microbiol. (2020) 77:1987–96. doi: 10.1007/s00284-020-02053-9, PMID: 32472262 PMC7415030

[218] More MI Swidsinski A. Saccharomyces Boulardii Cncm I-745 supports regeneration of the intestinal microbiota after diarrheic Dysbiosis - a review. Clin Exp Gastroenterol. (2015) 8:237–55. doi: 10.2147/CEG.S85574, PMID: 26316791 PMC4542552

[219] Kabbani TA Pallav K Dowd SE Villafuerte-Galvez J Vanga RR Castillo NE. Prospective randomized controlled study on the effects of Saccharomyces Boulardii Cncm I-745 and amoxicillin-Clavulanate or the combination on the gut microbiota of healthy volunteers. Gut Microbes. (2017) 8:17–32. doi: 10.1080/19490976.2016.1267890, PMID: 27973989 PMC5341914

